# Mediation and prognostic value of apparent diffusion coefficient in HCC patients receiving immune checkpoint inhibitors

**DOI:** 10.1186/s13244-025-02115-1

**Published:** 2025-10-25

**Authors:** Xiaona Fu, Yusheng Guo, Yanjie Yang, Shanshan Jiang, Bingxin Gong, Jie Lou, Lianwei Miao, Yixing Li, Peng Sun, Sichen Wang, Lixia Wang, Lian Yang

**Affiliations:** 1 Department of Radiology, Union Hospital, Tongji Medical College, Huazhong University of Science and Technology, Wuhan, China; 2 Hubei Provincial Clinical Research Center for Precision Radiology & Interventional Medicine, Wuhan, China; 3 Hubei Key Laboratory of Molecular Imaging, Wuhan, China; 4 Clinical & Technical Solutions, Philips Healthcare, Beijing, China; 5 School of Life Science and Technology, Computational Biology Research Center, Harbin Institute of Technology, Harbin, China

**Keywords:** Apparent diffusion coefficient, Mediation, Immune checkpoint inhibitors, Hepatocellular carcinoma, Prognosis

## Abstract

**Objectives:**

The study investigated the potential association between tumor size and inflammation from the standpoint of apparent diffusion coefficient (ADC) and the prognostic significance of ADC in hepatocellular carcinoma (HCC) patients treated with immune checkpoint inhibitors (ICIs).

**Materials and methods:**

This retrospective study ultimately included 255 HCC patients receiving ICIs, among whom 168 underwent posttreatment diffusion-weighted imaging at three months. The study analyzed the correlations among pretreatment tumor maximum diameter, inflammatory markers (neutrophil to lymphocyte ratio, NLR; platelet to lymphocyte ratio, PLR), and ADC (substantial tumor ADC, sADC; whole tumor ADC, wADC), as well as exploring the association between tumor maximum diameter and inflammatory markers regarding ADC. This study further focused on the prognostic value of baseline and relative change in ADC in HCC patients receiving ICIs.

**Results:**

The study found a potential relationship between tumor maximum diameter and inflammatory markers regarding sADC. Multivariate Cox regression showed that higher sADC was associated with longer overall survival (OS) (HR: 0.63, 95% CI: 0.43–0.91, *p* = 0.015). Additionally, the relative change in sADC (ΔsADC) positive group exhibited better progression-free survival (12 vs 6.4 months, *p* < 0.001) and OS (20.5 vs 13.3 months, *p* < 0.001) compared to the ΔsADC non-positive group, serving as an independent prognostic factor for HCC patients receiving ICIs.

**Conclusions:**

The association between tumor burden and inflammatory markers was observed regarding sADC, with baseline sADC and relative change promising as prognostic imaging biomarkers in HCC patients receiving ICIs.

**Critical relevance statement:**

The substantial tumor ADC is an important imaging feature revealing the potential association between tumor burden and inflammation, and its prognostic role for patients with HCC receiving ICIs.

**Key Points:**

- The sADC could indirectly reflect tumor microenvironment characteristics.
- The association between tumor burden and inflammatory markers was observed regarding sADC.
- Baseline sADC and its change predict prognosis in ICI-treated HCC patients.

**Graphical Abstract:**

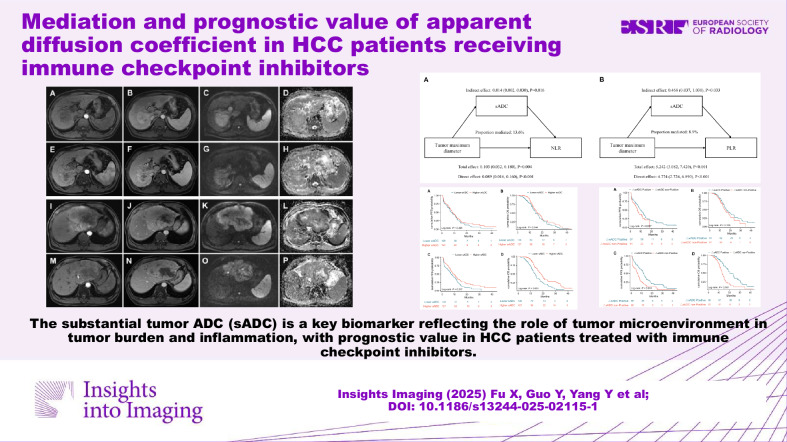

## Introduction

Hepatocellular carcinoma (HCC) represents the predominant form of primary liver malignancy, comprising approximately 75–85% of cases [1]. In recent years, immune checkpoint inhibitors (ICIs) have shown breakthrough progress in the treatment of advanced HCC [2]. ICIs exert antitumor effects by blocking the immunosuppressive signaling pathways, enabling the reactivation of effector T cells to mediate tumor cell killing [3]. However, a proportion of HCC patients develop primary or secondary resistance to ICIs and consequently fail to benefit from the treatment [4]. Therefore, identifying the sensitivity of ICIs is a major challenge and is crucial for optimizing therapeutic strategies in HCC.

Tumorigenesis and progression are associated with systemic inflammation, with the tumor microenvironment (TME) serving as a dynamic indicator of multiple tumor characteristics, such as cell density, tumor heterogeneity, and metabolic status, which frequently correlate closely with tumor burden [5]. In addition, structural and functional alterations of TME further facilitate the secretion of proinflammatory mediators and chemokines, thereby activating inflammatory cascades [6, 7]. These insights led us to hypothesize the potential mediating role of TME in tumor burden-inflammation interactions. Tumor size remains a conventional indicator of tumor biological behavior and progression, while neutrophil-lymphocyte ratio (NLR) and platelet-lymphocyte ratio (PLR) reflect the systemic inflammatory status [8, 9]. Multiparametric MRI has enabled non-invasive assessment of molecular and cellular features within the TME [10]. However, existing research has predominantly examined tumor size or inflammatory markers as independent prognostic factors [8, 11], the potential role of TME, indirectly assessed by noninvasive imaging techniques in the relationship between tumor size and inflammatory markers, has not been thoroughly investigated.

It was demonstrated that the TME not only regulates tumor growth, invasion, and metastasis but also has a profound impact on treatment response [12]. Diffusion-weighted imaging (DWI) is widely used to assess tumor activity by probing tumor physiology [13]. It explores the state of Brownian motion of water molecules in tissues via apparent diffusion coefficient (ADC), which indirectly captures the properties of the TME and provides important complementary information on the structure and viability of tissue cells [14–16]. The clinical utility of ADC is well-established in oncology. Zakaria et al [17] showed that compared to untreated cases, the ICIs treatment of brain metastasis was associated with a less invasive growth pattern, increased T-cell infiltration, and raised tumor ADC. Cuccarini et al [18] established that normalized minimum ADC in glioblastoma could predict immunotherapy response and survival outcomes while differentiating pseudoprogression from true progression. And Bao et al [19] suggested that ADC and ΔADC after neoadjuvant chemoimmunotherapy in resectable non-small cell lung cancer patients were effective biomarkers for predicting pathological response. Although multiple studies have investigated the prognostic value of ADC in HCC patients treated with radiotherapy, surgery, or ablation [20–22], its prognostic role in ICI-treated HCC remains underexplored, potentially due to heterogeneous immunotherapy responses and substantial treatment costs [23].

Based on the above research, this study aimed to investigate the potential association between tumor size and inflammation regarding ADC, while also exploring the prognostic role of ADC in HCC patients receiving ICIs. The first was to analyze the relationships among pretreatment tumor size, inflammatory markers (NLR and PLR), and ADC values, particularly assessing the potential role of ADC between tumor size and inflammatory markers. In addition, it was further explored whether ADC values and their changes could serve as sensitive biomarkers for predicting progression-free survival (PFS) and overall survival (OS) in HCC patients treated with ICIs.

## Methods and materials

### Patients

This retrospective study enrolled 255 HCC patients who received ICIs treatment at Union Hospital, Tongji Medical College, Huazhong University of Science and Technology, between July 2020 and December 2023, and was approved by the Research Ethics Committee of Tongji Medical College, Huazhong University of Science and Technology (Institutional Review Board No. S188). The inclusion criteria were as follows: (1) diagnosis of HCC based on the European Association for the Study of the Liver (EASL) guidelines [24], (2) Child–Pugh A or B, (3) Barcelona Clinic Liver Cancer (BCLC) stage B or C, (4) age 18 years, (5) receiving at least two cycles of immunotherapy, and (6) abdominal MRI enhancement prior to the first ICIs treatment. Exclusion criteria included: (1) severe cardiac, hepatic, or renal dysfunction or concurrent malignancies, (2) Child–Pugh class C, (3) BCLC stage A, and (4) incomplete clinical or imaging records. The study adhered to the Strengthening the Reporting of Observational Studies in Epidemiology (STROBE) guidelines [25]. As a retrospective analysis, patient-informed consent was waived. The study was approved by the local ethics committee and institutional review board.

### MRI procedure

MRI was performed on 3.0-T scanners (Magnetom Skyra or Magnetom Verio, Siemens Healthcare) equipped with multichannel phased-array coils. Following a 6–8 h fasting period, patients underwent abdominal imaging in the supine position. Fat-suppressed three-dimensional gradient-echo T1WI sequence and spin-echo T2WI sequence were acquired; DWI was acquired using echo planar imaging, and diffusion-encoded gradients with *b*-values of 50 s/mm^2^ and 800 s/mm^2^ were applied in the three orthogonal directions of the motion detection gradient. The contrast-enhanced sequence employed a three-dimensional volumetric interpolated breath-hold examination to obtain high-quality three-dimensional images quickly. Detailed scanning parameters are provided in Table S1.

### ADC measurement

All images were performed independently by two radiologists (with 5 years and 10 years of abdominal MRI experience, respectively) using picture archiving and communication system (PACS) workstations. The ADC maps were calculated automatically by the MRI system, and the ADC value was calculated by the following formula: $A{DC}=\frac{{\mathrm{ln}}({{\rm{SI}}}1/{{\rm{SI}}}2)}{({{\rm{b}}}2-{{\rm{b}}}1)}$, where SI1 and SI2 indicate the signal intensities acquired at *b* = b1 and *b* = b2 s/mm^2^, respectively. The site of the lesion was determined by contrast-enhanced MRI. Lesion localization was confirmed by contrast-enhanced MRI, with maximum tumor diameter measured in either arterial or portal venous phase on axial images. Both radiologists were informed of the HCC diagnosis, but not the clinical outcomes of the patients. Figure S1 shows the location diagram of ROIs. Five regions of interest (ROIs) of approximately 100 mm^2^ were manually outlined at the maximum layer of the tumor, excluding cysts, necrotic areas, and blood vessels, with the average value defined as the substantial tumor ADC (sADC). The ROIs manually outlined at the maximum layer of the tumor, including the entire tumor area (lesion, cysts, necrosis, and blood vessels), were defined as the whole tumor ADC (wADC). In addition, the relative ADC change was calculated as: $\varDelta A{DC}=\frac{({{\rm{ADC}}}\; {{\rm{at}}}\,3\,{{\rm{months}}}\,-\,{{\rm{ADC}}}\; {{\rm{baseline}}})}{{{\rm{ADC}}}\; {{\rm{baseline}}}}$. An elevation in ΔADC was categorized as the ΔADC positive group, while no change or a decrease in ΔADC values was categorized as the ΔADC non-positive group.

### Data collection and follow-up

All baseline data were obtained from patients’ electronic medical records. Tumor responses were assessed according to the modified Response Evaluation Criteria in Solid Tumors (mRECIST) guidelines, including complete response (CR), partial response (PR), stable disease (SD), or disease progression (PD). The sum of CR and PR was the objective remission rate (ORR), and the sum of CR, PR, and SD was the disease control rate (DCR). PFS was the time between initial immunotherapy treatment and tumor progression or patient death, however, OS was the time between initial immunotherapy treatment and the endpoint of the last follow-up or death.

### Statistical analysis

Continuous variables were assessed for normality, with normally distributed data presented as mean (SD) and non-normally distributed data as median (IQR). Categorical variables were expressed as counts (%). The intraclass correlation coefficient (ICC) was used to assess the agreement of ADC measurements between two radiologists. Spearman correlation analyses were performed for tumor maximum diameter, inflammatory markers (NLR and PLR), and ADC values (wADC and sADC) prior to treatment with ICIs, correcting for age and sex. In order to reduce the probability of type I error due to multiple comparisons, we applied Benjamini–Hochberg false discovery rate (FDR) correction that adjusted the threshold of statistical significance to a *P*_FDR_ < 0.05. To provide additional clinical insights, the ADC threshold was determined by the Youden’s index of the receiver operating characteristic (ROC) curve of response to treatment.

In mediation analyses, tumor maximum diameter was defined as the predictor (X), wADC and sADC were defined as the mediators (M), and NLR and PLR were defined as the outcome (Y). All analyses were corrected for age and sex. Mediation analyses were performed using an available R package named “mediation” [26]. The threshold values for wADC and sADC are based on their median, respectively. The Kaplan–Meier curves and Log-rank *p* were used to compare the differences in PFS and OS between the groups. Variables with *p* < 0.1 in the univariate analysis were included in the multivariate Cox regression model to calculate the hazard ratios for PFS and OS. Forest plots showed PFS and OS hazard ratios for each subgroup. The tests were two-tailed, and *p* < 0.05 was considered statistically significant. All analyses in this study were performed using SPSS version 26.0 software (IBM, Armonk, NY, USA) and R version 4.3.0 (R Foundation).

## Results

### Demographic and clinical characteristics of patients

Table 1 showed the baseline demographic and clinical characteristics of all patients, as well as those who had DWI images after three months of ICIs treatment. A total of 255 HCC patients treated with ICIs were included in this study, comprising 221 males (86.7%) and 34 females (13.3%). BCLC stage revealed that 86 patients (33.7%) had stage B, while 169 (66.3%) were in stage C. Of the 168 individuals with DWI images at three months after ICI treatment, baseline characteristics included 147 males (87.5%) and 21 (12.5%) females. The median maximum diameter of HCC before ICIs treatment was 6.65 cm, and the number of tumors > 3 was observed in 38 patients (22.6%).

**Table 1:** Baseline characteristics of patients

Characteristics	Total population (*n* = 255)	Population with DWI after three months of ICIs treatment (*n* = 168)
Age (years), median (IQR)	58 (50, 64)	58 (50, 64)
Sex, *n* (%)		
Female	34 (13.3%)	21 (12.5%)
Male	221 (86.7%)	147 (87.5%)
BMI (kg/m^2^), mean (SD)	22.9 (3.1)	22.8 (3.1)
Types of ICIs, *n* (%)		
PD-1	241 (94.5%)	157 (93.5%)
PD-L1	14 (5.5%)	11 (6.5%)
Tumor maximum diameter (cm), median (IQR)	6.7 (3.9, 10.1)	6.7 (3.8, 9.9)
Tumor number, *n* (%)		
≤ 3	186 (72.9%)	130 (77.4%)
> 3	69 (27.1%)	38 (22.6%)
PVTT, *n* (%)	103 (40.4%)	77 (45.8%)
Lymph node metastasis, *n* (%)	67 (26.3%)	48 (28.6%)
BCLC stage, *n* (%)		
B	86 (33.7%)	51 (30.4%)
C	169 (66.3%)	117 (69.6%)
Child–Pugh, *n* (%)		
A	219 (85.9%)	149 (88.7%)
B	36 (14.1%)	19 (11.3%)
ECOG PS, *n* (%)		
0	178 (69.8%)	129 (76.8%)
≥ 1	77 (30.2%)	39 (23.2%)
HBV infection, *n* (%)	137 (53.7%)	82 (48.8%)
Smoking, *n* (%)	62 (24.3%)	40 (23.8%)
Drinking, *n* (%)	36 (14.1%)	26 (15.5%)
NLR, median (IQR)	2.5 (1.6, 3.6)	2.6 (1.6, 3.8)
PLR, median (IQR)	120.5 (87.3, 177.5)	130 (89.8, 184.3)
ALBI, mean (SD)	−2.41 (0.47)	−2.46 (0.43)
Hemoglobin (g/L), median (IQR)	132 (120.5, 142.5)	132 (121, 142)
ALT(U/L), median (IQR)	34 (22, 55.5)	34 (22.8, 57.5)
AST(U/L), median (IQR)	43 (30, 71.5)	42 (29, 71)
AFP level (ng/mL), *n* (%)		
< 400	161 (63.1%)	108 (64.3%)
≥ 400	94 (36.9%)	60 (35.7%)

*BMI* body mass index, *ICIs* immune checkpoint inhibitors, *PVTT* portal vein tumor thrombosis, *BCLC* Barcelona Clinic Liver Cancer, *ECOG PS* Eastern Cooperative Oncology Group Performance Status, *HBV infection* hepatitis B virus infection, *NLR* neutrophil to lymphocyte ratio, *PLR* platelet to lymphocyte ratio, *ALBI* albumin–bilirubin, *ALT* alanine transaminase, *AST* aspartate transaminase, *AFP* alpha-fetoprotein

### Threshold and changes in ADC

**Fig. 1:**
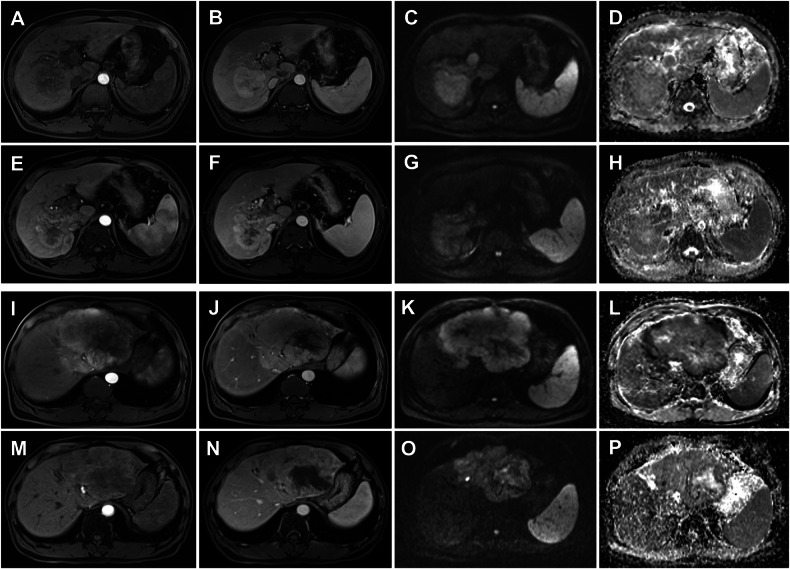
Images and groups of two HCC patients before and after three months of ICIs treatment. A 60-year-old male patient’s arterial phase (**A**, **E**), venous phase (**B**, **F**), high *b*-value DWI (**C**, **G**), and ADC (**D**, **H**) maps before and three months after treatment, respectively. Before treatment, wADC = 1605.61 × 10^−6^ mm²/s and sADC = 1308.99 × 10^−6^ mm²/s, after treatment, wADC = 1477.99 × 10^−6^ mm²/s and sADC = 1159.98 × 10^−6^ mm²/s. This patient was categorized into the higher wADC group, the higher sADC group, the ΔwADC non-positive group, and the ΔsADC non-positive group. A 59-year-old male patient’s arterial phase (**I**, **M**), venous phase (**J**, **N**), high *b*-value DWI (**K**, **O**), and ADC (**L**, **P**) maps before and three months after treatment, respectively. Before treatment, wADC = 507.37 × 10^−6^ mm²/s and sADC = 443.94 ×10^−6^ mm²/s, after treatment, wADC = 1336.62 × 10^−6^ mm²/s and sADC = 1327.94 × 10^−6^ mm²/s. This patient was categorized into the lower wADC group, the lower sADC group, the ∆wADC positive group, and the ∆sADC positive group. HCC, hepatocellular carcinoma; ICIs, immune checkpoint inhibitors; wADC, whole tumor apparent diffusion coefficient; sADC, substantial tumor apparent diffusion coefficient; ΔwADC, relative change in whole tumor apparent diffusion coefficient; ΔsADC, relative change in substantial tumor apparent diffusion coefficient

Pretreatment tumor ADC values demonstrated excellent interobserver agreement (wADC, ICC = 0.91; 95% CI: 0.89–0.93; sADC, ICC = 0.91; 95% CI: 0.89–0.93), which persisted at 3-month follow-up (ΔwADC, ICC = 0.94; 95% CI: 0.92–0.96; ΔsADC, ICC = 0.93; 95% CI: 0.91–0.95). The median pretreatment wADC was 1144.63 × 10^−6^ mm²/s, with 128 patients included in the lower wADC group and 127 in the higher wADC group. And pretreatment sADC had a median of 948.07 × 10^−6 ^mm²/s, with 128 patients included in the Lower sADC group and 127 in the Higher sADC group. Among the 168 who had DWI images three months after ICI treatment, 87 patients were classified into the ΔwADC positive group, and 81 patients into the ΔwADC non-positive group based on the change of wADC. Similarly, according to the change of sADC, 86 and 82 patients were categorized into the ΔsADC Positive and ΔsADC-non positive groups, respectively. Figure 1 demonstrated the images and groups of two HCC patients before and three months after treatment with ICIs. To provide additional clinical insights, the patients were categorized into Response and Non-response groups by the ADC threshold based on PR (wADC, 1253.4 × 10^−6^ mm²/s, sADC, 887.54 × 10^−6^ mm²/s). The patients were categorized into control and non-control groups based on the ADC threshold determined by a combination of PR and SD (PR + SD) (wADC, 1041.2 × 10^−6^ mm²/s, sADC, 898.42 × 10^−6^ mm²/s).

### Correlation analyses among tumor maximum diameter, ADC values, and inflammatory markers

**Fig. 2:**
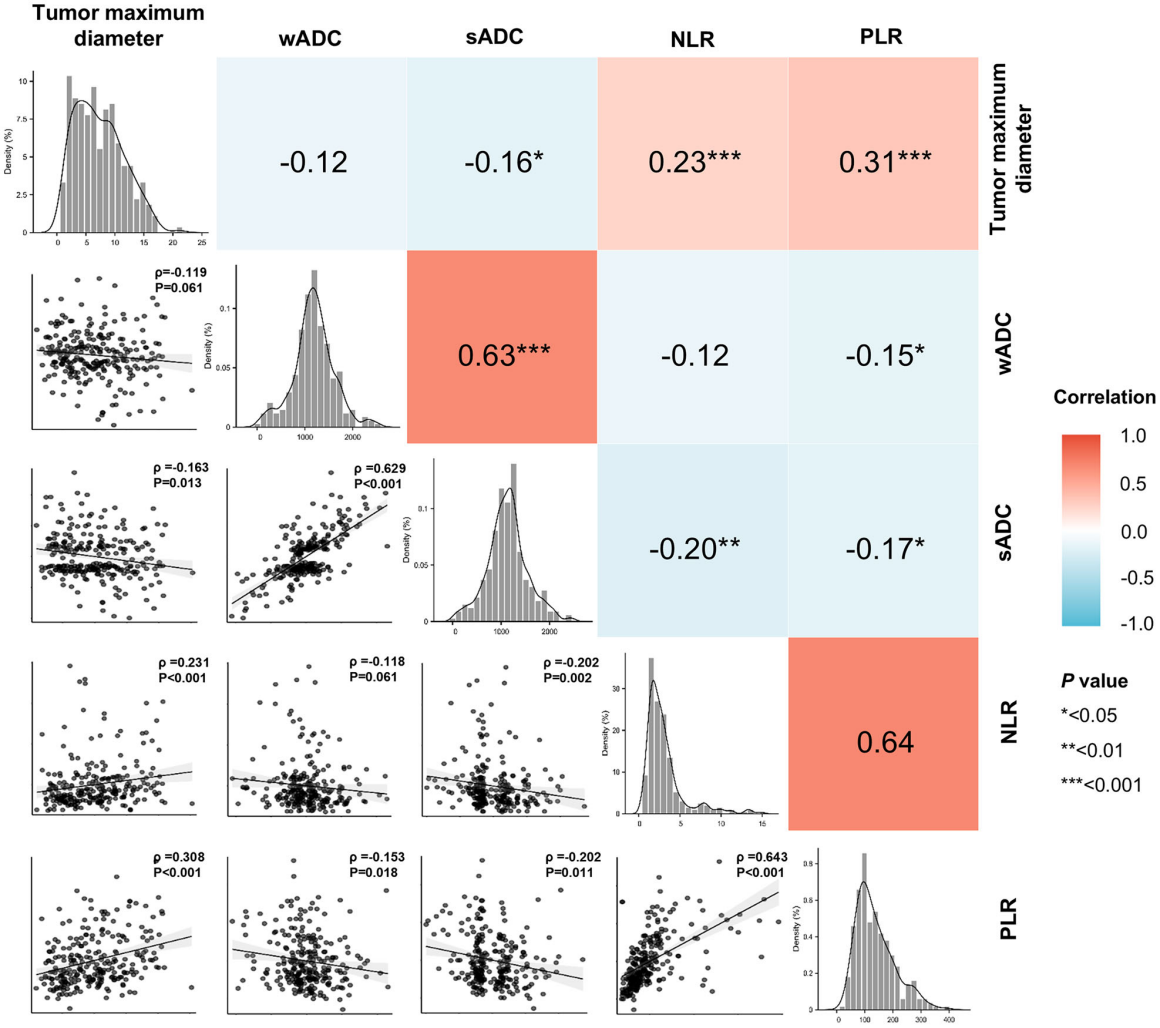
Correlation analyses of tumor maximum diameter, ADC values, and inflammatory markers adjusted for age and sex. wADC, whole tumor apparent diffusion coefficient; sADC, substantial tumor apparent diffusion coefficient; NLR, Neutrophil to lymphocyte ratio; PLR, Platelet to lymphocyte ratio

**Fig. 3:**
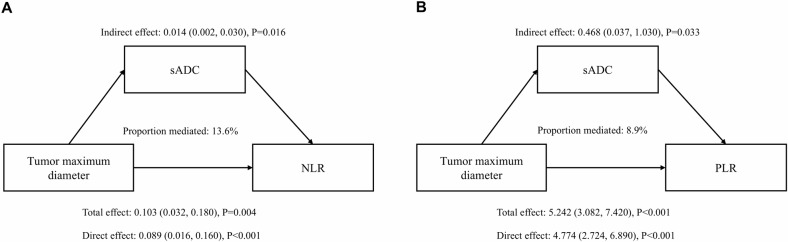
Mediation effects of sADC on the association between tumor maximum diameter and NLR (**A**) and PLR (**B**), adjusted for age and sex. sADC, substantial tumor apparent diffusion coefficient; NLR, neutrophil to lymphocyte ratio; PLR, platelet to lymphocyte ratio

According to the FDR corrected correlation analyses (Fig. 2), tumor maximum diameter was significantly negatively correlated with sADC (*p* = 0.013) and positively correlated with NLR (*p* < 0.001) and PLR (*p* < 0.001). sADC had a significant negative correlation with NLR (*p* = 0.002) and PLR (*p* = 0.011). Mediation analyses demonstrated that the potential relationship between tumor maximum diameter and NLR (total effect = 0.103, *p* = 0.004, direct effect = 0.089, *p* < 0.001, indirect effect = 0.014, *p* = 0.016, Fig. 3A) and PLR (total effect = 5.242, *p* < 0.001, direct effect = 4.774, *p* < 0.001, indirect effect = 0.468, *p* = 0.033, Fig. 3B) regarding sADC. However, the wADC did not reveal a significant potential association of tumor maximum diameter with NLR and PLR (Tables S2 and S3).

### Tumor response

There were no significant differences in ORR and DCR between the lower wADC and higher wADC groups (Table S4). The Higher sADC group had a greater DCR than the Lower sADC group, but the ORR was not significantly different between the two groups (Table S5). Among patients with post-treatment DWI, there was no statistically significant difference in ORR (*p* = 0.087) and DCR (*p* = 0.320) in the ΔwADC Positive group and ΔwADC non-Positive group (Table S6). The ORR (*p* = 0.002, Table S7) and DCR (*p* = 0.002) were significantly higher in the ΔsADC Positive group compared to the ΔsADC non-Positive group.

### Survival analysis

**Fig. 4:**
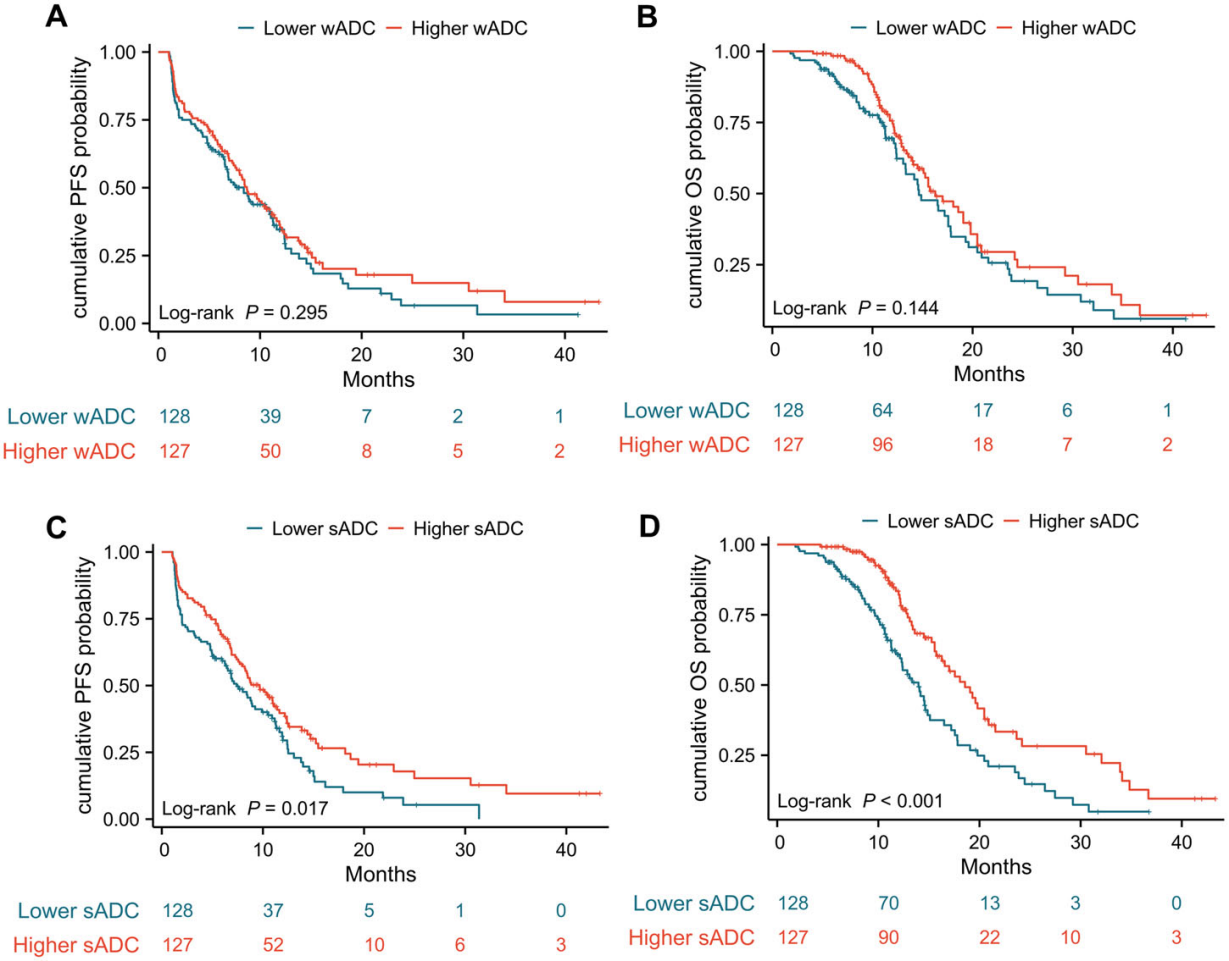
Kaplan–Meier curves for PFS (**A**, **C**) and OS (**B**, **D**) for the Lower wADC and Higher wADC groups, as well as for the Lower sADC and Higher sADC groups. PFS, progression-free survival; OS, overall survival; wADC, whole tumor apparent diffusion coefficient; sADC, substantial tumor apparent diffusion coefficient

**Fig. 5:**
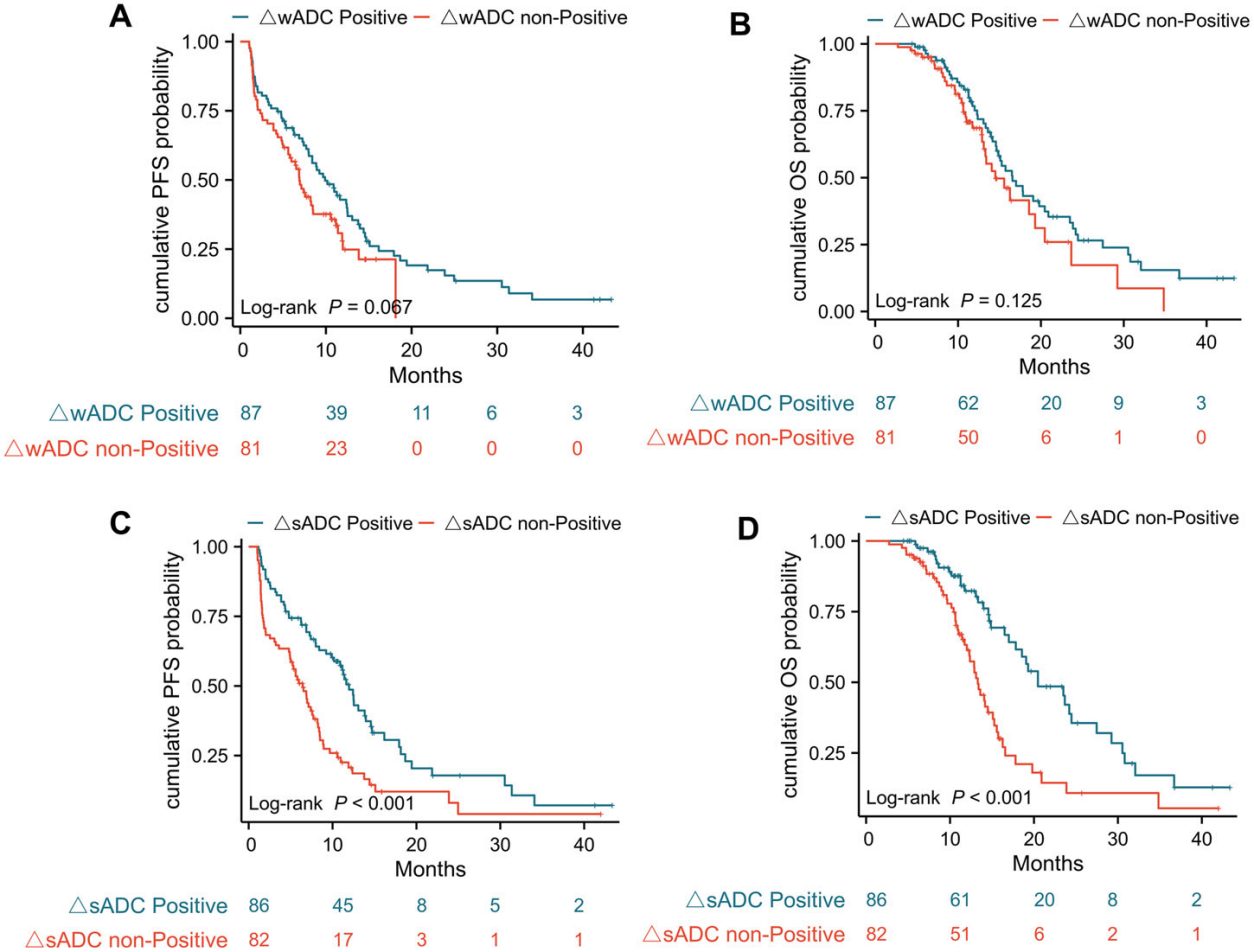
Kaplan–Meier curves for PFS (**A**, **C**) and OS (**B**, **D**) for the ΔwADC Positive and ΔwADC non-Positive groups, as well as for the ΔsADC Positive and ΔsADC non-Positive groups. PFS, progression-free survival; OS, overall survival; ΔwADC, the relative change in whole tumor apparent diffusion coefficient; ΔsADC, the relative change in substantial tumor apparent diffusion coefficient

The median follow-up duration was 15.73 months (95% CI: 14.5–18.07) for the entire cohort and 15.73 months (95% CI: 14.5–19.3) for the 168 patients with post-treatment DWI. KM curves indicated no significant differences in PFS (*p* = 0.295) and OS (*p* = 0.144) between Higher and Lower wADC groups (Fig. 4). However, Higher sADC showed longer PFS (9.7 vs 7.4 months, *p* = 0.017) and OS (18.6 vs 14 months, *p* < 0.001) compared to Lower sADC. For those treated with ICIs for 3 months, no significant PFS (*p* = 0.067) and OS (*p* = 0.125) differences were observed between ΔwADC groups (Fig. 5). In contrast, the ΔsADC positive group had significantly longer PFS (12 vs 6.4 months, *p* < 0.001) and OS (20.5 vs 13.3 months, *p* < 0.001).

Variables demonstrating potential significance (*p* < 0.1) in the univariate analysis were subsequently included in the multivariate regression. The multivariate analyses showed that smoking and high AFP levels were independent risk factors for shorter PFS in pretreatment 255 patients (Table S8). Meanwhile, tumor maximum diameter, ECOG PS, ALT, AFP level, and sADC were independent predictors of OS in patients (Table S9). Multivariate analyses of patients with DWI images after three months of HCC receiving ICIs showed that smoking and high AFP level were independent risk factors for shorter PFS (Table 2), while ΔsADC Positive was an independent protective factor for PFS. In addition, Tumor maximum diameter, ECOG PS, ALT, AFP level, and ΔsADC were also independent predictors of OS in patients (Table 3).

**Table 2:** Univariate and multivariate Cox proportional hazards analyses for PFS in patients with DWI three months after ICIs treatment

Characteristics	Univariate analysis		Multivariate analysis	
	Hazard ratio (95% CI)	*p* value	Hazard ratio (95% CI)	*p* value
Age (years)	0.994 (0.979–1.009)	0.411		
Sex				
Female	Reference			
Male	0.709 (0.429–1.171)	0.179		
BMI (kg/m^2^)	1.024 (0.967–1.085)	0.413		
Types of ICIs				
PD-1	Reference		Reference	
PD-L1	**0.498 (0.231–1.073)**	**0.075**	0.507 (0.234–1.096)	0.084
Tumor maximum diameter (cm)	1.029 (0.989–1.071)	0.155		
Tumor number				
≤ 3	Reference			
> 3	0.924 (0.602–1.419)	0.718		
PVTT				
No	Reference			
Yes	0.995 (0.697–1.420)	0.976		
Lymph node metastasis				
No	Reference			
Yes	1.055 (0.715–1.558)	0.787		
BCLC stage				
B	Reference			
C	1.332 (0.885–2.005)	0.170		
Child–Pugh				
A	Reference			
B	0.992 (0.545–1.806)	0.980		
ECOG PS				
0	Reference			
≥ 1	1.299 (0.849–1.987)	0.229		
HBV infection				
No	Reference			
Yes	0.846 (0.594–1.206)	0.355		
Smoking				
No	Reference		Reference	
Yes	1.513 (1.017–2.250)	**0.041**	1.533 (1.029–2.283)	**0.036**
Drinking				
No	Reference			
Yes	1.310 (0.823–2.085)	0.255		
NLR	0.968 (0.899–1.042)	0.385		
PLR	1.001 (0.998–1.003)	0.555		
ALBI	0.904 (0.586–1.396)	0.650		
Hemoglobin (g/L)	0.994 (0.983–1.005)	0.285		
ALT (U/L)	1.002 (0.999–1.005)	0.225		
AST (U/L)	1.000 (0.999–1.002)	0.527		
AFP level (ng/mL)				
< 400	Reference		Reference	
≥ 400	1.546 (1.075–2.222)	**0.019**	1.543 (1.069–2.227)	**0.021**
ΔwADC group				
ΔwADC non-positive	Reference		Reference	
ΔwADC Positive	0.704 (0.484–1.026)	**0.068**	0.791 (0.537–1.165)	0.235
ΔsADC group				
ΔsADC non-positive	Reference		Reference	
ΔsADC positive	0.500 (0.349–0.717)	**< 0.001**	0.530 (0.365–0.769)	**< 0.001**

Significant values are given in bold

*PFS* progression-free survival, *BMI* body mass index, *ICIs* immune checkpoint inhibitors, *PVTT* portal vein tumor thrombosis, *BCLC* Barcelona Clinic Liver Cancer, *ECOG PS* Eastern Cooperative Oncology Group Performance Status, *HBV* *infection* hepatitis B virus infection, *NLR* neutrophil to lymphocyte ratio, *PLR* platelet to lymphocyte ratio, *ALBI* albumin–bilirubin, *ALT* alanine transaminase, *AST* aspartate transaminase, *AFP* alpha-fetoprotein, *ΔwADC* relative change in whole tumor apparent diffusion coefficient, *ΔsADC* relative change in substantial tumor apparent diffusion coefficient, *CI* confidence interval

**Table 3:** Univariate and multivariate Cox proportional hazards analyses for OS in three months after ICIs treatment in patients with DWI

Characteristics	Univariate analysis		Multivariate analysis	
	Hazard ratio (95% CI)	*p* value	Hazard ratio (95% CI)	*p* value
Age (years)	0.996 (0.978–1.015)	0.711		
Sex				
Female	Reference			
Male	1.632 (0.814–3.270)	0.168		
BMI (kg/m^2^)	1.004 (0.935–1.078)	0.919		
Types of ICIs				
PD-1	Reference			
PD-L1	0.609 (0.246–1.506)	0.283		
Tumor maximum diameter (cm)	1.051 (1.000–1.103)	**0.048**	1.061 (1.008–1.116)	**0.024**
Tumor number				
≤ 3	Reference			
> 3	0.729 (0.422–1.258)	0.256		
PVTT				
No	Reference			
Yes	1.136 (0.736–1.753)	0.564		
Lymph node metastasis				
No	Reference			
Yes	0.671 (0.411–1.097)	0.112		
BCLC stage				
B	Reference			
C	1.158 (0.692–1.936)	0.576		
Child–Pugh				
A	Reference			
B	1.162 (0.559–2.414)	0.688		
ECOG PS				
0	Reference		Reference	
≥ 1	1.822 (1.067–3.112)	**0.028**	2.202 (1.267–3.827)	**0.005**
HBV infection				
No	Reference			
Yes	0.944 (0.615–1.449)	0.792		
Smoking				
No	Reference			
Yes	1.316 (0.818–2.115)	0.257		
Drinking				
No	Reference			
Yes	0.635 (0.343–1.176)	0.148		
NLR	0.992 (0.913–1.078)	0.858		
PLR	1.001 (0.998–1.004)	0.463		
ALBI	0.958 (0.547–1.676)	0.879		
Hemoglobin (g/L)	0.993 (0.980–1.006)	0.310		
ALT(U/L)	1.005 (1.002–1.008)	**0.003**	1.005 (1.001–1.008)	**0.009**
AST(U/L)	1.001 (1.000–1.002)	0.140		
AFP level (ng/mL)				
< 400	Reference		Reference	
≥ 400	2.004 (1.303–3.084)	**0.002**	2.235 (1.439–3.473)	**< 0.001**
ΔwADC group				
ΔwADC non-positive	Reference			
ΔwADC positive	0.703 (0.448–1.104)	0.126		
ΔsADC group				
ΔsADC non-positive	Reference		Reference	
ΔsADC positive	0.428 (0.274–0.667)	**< 0.001**	0.372 (0.236–0.587)	**< 0.001**

Significant values are given in bold

*OS* overall survival, *BMI* body mass index, *ICIs* immune checkpoint inhibitors, *PVTT* portal vein tumor thrombosis, *BCLC* Barcelona Clinic Liver Cancer, *ECOG PS* Eastern Cooperative Oncology Group Performance Status, *HBV infection* hepatitis B virus infection, *NLR* neutrophil to lymphocyte ratio, *PLR* platelet to lymphocyte ratio, *ALBI* albumin–bilirubin, *ALT* alanine transaminase, *AST* aspartate transaminase, *AFP* alpha-fetoprotein, *ΔwADC* relative change in whole tumor apparent diffusion coefficient, *ΔsADC* relative change in substantial tumor apparent diffusion coefficient, *CI* confidence interval

To provide additional clinical insights, the wADC thresholds were determined according to PR or (PR + SD), respectively, and there were no significant differences found in PFS and OS between groups (all *p* values, *p* > 0.05, Fig. S2). The response group showed longer PFS (9.2 vs 6.4 months, *p* = 0.035, Fig. S3A) and OS (16.6 vs 14.2 months, *p* = 0.041, Fig. S3B) than the non-response group according to the sADC threshold determined by PR. And the control group had longer PFS (9.2 vs 6.4 months, *p* = 0.040, Fig. S3C) and OS (16.6 vs 14.2 months, *p* = 0.035, Fig. S3D) than the non-control group according to the sADC threshold determined by PR + SD.

### Subgroup analysis

Subgroup analyses of both the pretreatment cohort (*n* = 255) and posttreatment cohort (*n* = 168) showed relatively consistent results in PFS and OS. Figures S4 and S5 showed the effect of wADC on the survival of different subgroups, respectively. The Higher sADC group was associated with a lower risk of short PFS in subgroups of males, PD-1 usage, without PVTT, no lymph node metastasis, Child–Pugh A, ECOG PS ≥ 1, without hepatitis B virus (HBV) infection, and no smoking (Fig. S6). The Higher sADC group had a lower risk of short OS, with exceptions in the subgroups of female, PD-L1 usage, with PVTT, with lymph node metastasis, Child–Pugh B, smoking, and AFP ≥ 400 ng/mL (Fig. S7). In the posttreatment analysis of 168 individuals, Figs. S8 and S9 showed the effect of ΔwADC on the survival of different subgroups, respectively. Except for the subgroups with PD-L1 usage, tumor number > 3, Child–Pugh B, ECOG PS ≥ 1, and drinking, the ΔsADC positive group showed a lower risk of short PFS than the ΔsADC non-positive group (Fig. S10). The risk of short OS in the ΔsADC positive group was lower than that in the ΔsADC non-positive group, except for the subgroups of age ≥ 60, female, PD-L1 usage, tumor number > 3, without PVTT, BCLC-B stage, ECOG PS ≥ 1, no HBV infection, and AFP < 400 ng/mL (Fig. S11).

## Discussion

This study was the first to investigate the potential association between tumor size and inflammation regarding ADC, as well as the prognostic impact of ADC in HCC patients treated with ICIs. The study revealed that the potential association between tumor maximum diameter and inflammatory markers before ICIs treatment regarding sADC. Further analyses showed that patients in the Higher sADC group were associated with longer OS, and ΔsADC could serve as an independent predictor of OS and PFS. These findings not only provide novel insights into tumor-inflammation interaction, but also provide a new imaging basis for ICIs personalized treatment strategy for HCC through sADC and its relative changes.

Liver cancer is a common global cancer, with the TME playing a crucial role in its development and treatment response [12]. DWI measures water molecule diffusion in tissues using the ADC, reflecting cell density and tumor activity. This study found the potential relationship between tumor size and NLR and PLR regarding sADC, especially NLR. It is possible that different tumor sizes presented different TME structures or functional states. Larger tumors may induce more significant TME structural changes (e.g., increased cell density) or TME functional adaptations such as significant stromal remodeling and hypoxia, promoting proinflammatory mediator release and systemic inflammation [7, 27, 28]. NLR primarily reflects local immune activity, with neutrophil and lymphocyte fluctuations being directly influenced by the tumor inflammatory microenvironment, including immune cell infiltration and regional cytokine production [29]. Tumor ADC values, reflecting tissue microstructure characteristics, may modulate NLR by regulating immune cell recruitment and activation. In contrast, PLR is more reflective of systemic hemodynamics and coagulation mechanisms. While platelets contribute significantly to tumor-associated angiogenesis, metastasis, and coagulation, their activation depends not only on the tumor environment [30] but also on systemic circulatory and circulatory dynamics. Tumor ADC describes tissue properties and, therefore, may not fully capture changes in these systemic mechanisms. These findings offer mechanistic insights into how tumor burden may influence systemic inflammation via TME modulation. However, this study only focused on the application of ADC in patients, and further exploration of the underlying biological mechanisms combining multiple sequence parameters and histopathological features is required in the future.

**Fig. 6:**
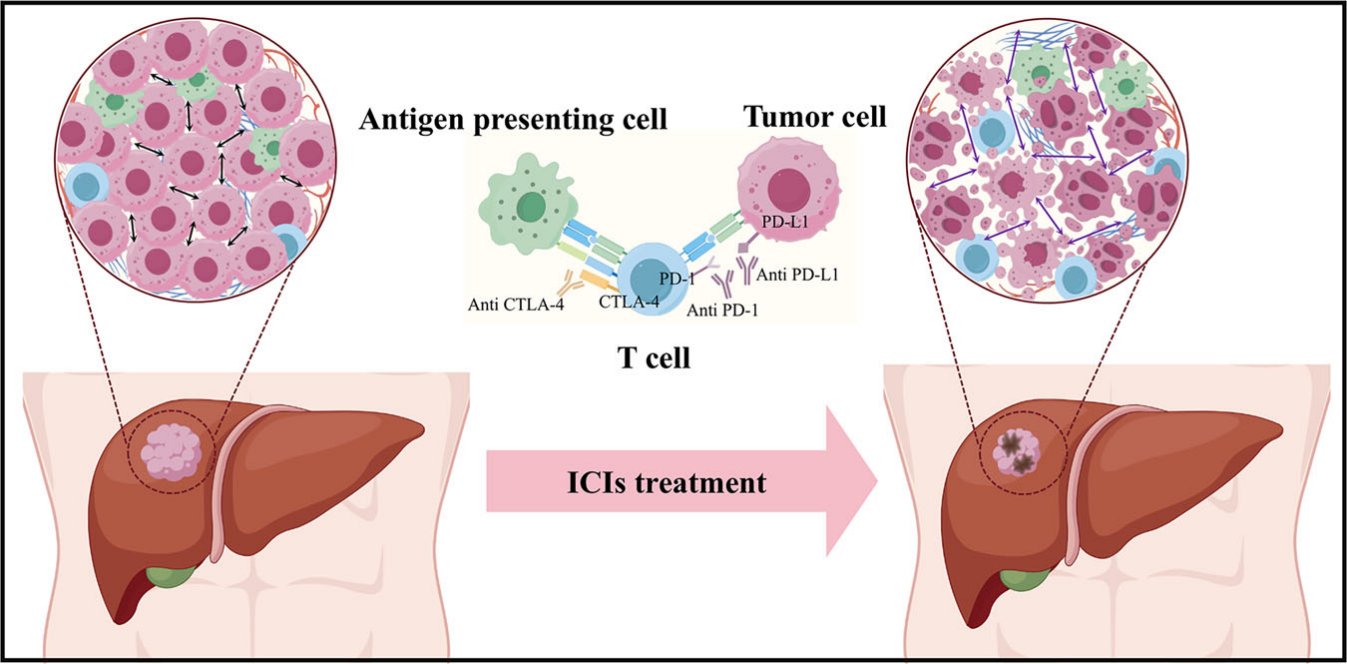
The schematic of water molecules diffusion with effective ICIs treatment. The black and purple arrows represent the diffusion capacity of water molecules before and after treatment, respectively

With advancements in treatment technologies, ICIs have become vital for advanced HCC [2]. However, treatment resistance causes heterogeneous ICI responses [4]. This study found that higher sADC values were associated with longer OS. This correlation could be attributed to the fact that higher sADC indicated freer water diffusion in tumor tissue, suggesting less active and malignant tumors that respond better to ICIs. Previous study demonstrated that optimal baseline ADC had the ability to predict tumor response and prolonged survival after chemoembolization in HCC patients [31]. The present study also found that the relative increase in sADC favored the prognosis of HCC patients treated with ICIs, similar to previous studies. Yu et al found that dynamic changes in ADC values of radiation therapy in HCC patients were significantly associated with local PFS [20]. The prognostic utility of ADC dynamics extends beyond HCC, having demonstrated reliable prediction of treatment response in various malignancies, including chemotherapy outcomes for unresectable pancreatic cancer [20] and early therapeutic evaluation in chemoradiotherapy for head and neck and cervical cancers [32, 33]. In addition, we showed that in most subgroups, the ∆sADC-Positive group had a lower risk of short PFS or OS than the ∆sADC non-Positive group, providing insights into how ∆sADC affects survival across various subgroups. Therefore, clinicians could measure sADC before and three months after ICIs treatment at the level of maximum tumor diameter. A decreased sADC may indicate a poorer prognosis, potentially identifying patients unlikely to benefit from ICIs and enabling timely treatment modification. The Fig. 6 showed the diffusion of water molecules with effective ICIs treatment in HCC patients. This may arise from the phenomenon that the biological behavior of tumors changes during treatment. The relative increase of sADC may reflect treatment-induced tumor cell death or apoptosis, resulting in enhanced diffusion of water molecules, which is strongly associated with improved treatment efficacy [34, 35].

The study also found that elevated ALT and AFP levels were independent risk factors for reduced OS in HCC patients receiving ICIs, consistent with previous research. [36, 37]. ALT is an enzyme primarily in hepatocytes, and HCC induces hepatocytolysis by destroying liver tissue, which leads to the release of ALT [38]. Elevated ALT levels suggested greater liver damage, potentially decreasing treatment effectiveness and shortening OS. In addition, AFP has been widely used to monitor the progression of the disease [39], with elevated concentrations correlating with greater tumor burden, more advanced disease stages, and higher biological aggressiveness, potentially reducing immunotherapy effectiveness and worsening patient prognosis.

There were some limitations of our study. First, it was a single-center retrospective study with a relatively small patient sample. Therefore, a multicenter prospective study with a large sample is needed to further validate our results. Methodologically, the sADC and wADC in this study were localized based on the tumor maximum diameter layer. In recent years, there have also been several studies exploring the value of histogram and volumetric analyses of ADC in oncology. Future studies should incorporate more comprehensive quantitative methods, such as histogram analysis or three-dimensional ROI measurements, to enhance measurement accuracy and result in generalizability. In addition, we performed follow-up DWI at 3 months after initiating treatment, but as an early marker for predicting treatment response, future studies may try to perform DWI at 1–2 months after treatment to assess whether treatment response can be captured earlier.

## Conclusions

The study uncovered that potential relationship between maximum tumor diameter and inflammatory markers (NLR and PLR) before ICIs treatment regarding sADC, possibly suggesting a potential mechanism by which tumor burden affects systemic inflammation via the TME. Additionally, baseline sADC and its changes may serve as imaging biomarkers for predicting the prognosis of HCC patients receiving ICIs.

## Supplementary information

ELECTRONIC SUPPLEMENTARY MATERIAL

## Data Availability

The datasets used and/or analyzed in this study are available from the corresponding author on reasonable request.

## Abbreviations

ADC: Apparent diffusion coefficient
CR: Complete response
DCR: Disease control rate
DWI: Diffusion-weighted imaging
HCC: Hepatocellular carcinoma
ICC: Intraclass correlation coefficients
ICIs: Immune checkpoint inhibitors
NLR: Neutrophil to lymphocyte ratio
ORR: Objective remission rate
OS: Overall survival
PD: Disease progression
PFS: Progression-free survival
PLR: Platelet to lymphocyte ratio
PR: Partial response
sADC: Substantial tumor ADC
SD: Stable disease
TME: Tumor microenvironment
wADC: Whole tumor ADC
ΔsADC: Relative change in substantial tumor apparent diffusion coefficient
ΔwADC: Relative change in whole tumor apparent diffusion coefficient

## References

[1] Zhou J, Sun H, Wang Z et al (2023) Guidelines for the diagnosis and treatment of primary liver cancer (2022 edition). Liver Cancer 12:405–444 37901768 10.1159/000530495 PMC10601883

[2] Cheng AL, Hsu C, Chan SL, Choo SP, Kudo M (2020) Challenges of combination therapy with immune checkpoint inhibitors for hepatocellular carcinoma. J Hepatol 72:307–319 31954494 10.1016/j.jhep.2019.09.025

[3] Sangro B, Sarobe P, Hervas-Stubbs S, Melero I (2021) Advances in immunotherapy for hepatocellular carcinoma. Nat Rev Gastroenterol Hepatol 18:525–543 33850328 10.1038/s41575-021-00438-0 PMC8042636

[4] Pinter M, Jain RK, Duda DG (2021) The current landscape of immune checkpoint blockade in hepatocellular carcinoma: a review. JAMA Oncol 7:113–123 33090190 10.1001/jamaoncol.2020.3381 PMC8265820

[5] Xu J, Xu X, Zhang H, Wu J, Pan R, Zhang B (2024) Tumor-associated inflammation: the role and research progress in tumor therapy. J Drug Deliv Sci Tec 102:106376

[6] Chen Z, Han F, Du Y, Shi H, Zhou W (2023) Hypoxic microenvironment in cancer: molecular mechanisms and therapeutic interventions. Signal Transduct Target Ther 8:70 36797231 10.1038/s41392-023-01332-8 PMC9935926

[7] Marozzi M, Parnigoni A, Negri A et al (2021) Inflammation, extracellular matrix remodeling, and proteostasis in tumor microenvironment. Int J Mol Sci 22:8102 34360868 10.3390/ijms22158102 PMC8346982

[8] Yin Y, Wang W, Tang M, Liu W (2024) Investigating the impact of tumor size on survival outcomes in thymoma and thymic carcinoma patients using the SEER database. Sci Rep 14:27680 39533067 10.1038/s41598-024-79186-5 PMC11557901

[9] Ji JH, Ha SY, Lee D et al (2023) Predictive biomarkers for immune-checkpoint inhibitor treatment response in patients with hepatocellular carcinoma. Int J Mol Sci 24:7640 37108802 10.3390/ijms24087640 PMC10144688

[10] Hoffmann E, Masthoff M, Kunz WG et al (2024) Multiparametric MRI for characterization of the tumour microenvironment. Nat Rev Clin Oncol 21:428–448 38641651 10.1038/s41571-024-00891-1

[11] Dharmapuri S, Özbek U, Lin JY et al (2020) Predictive value of neutrophil to lymphocyte ratio and platelet to lymphocyte ratio in advanced hepatocellular carcinoma patients treated with anti-PD-1 therapy. Cancer Med 9:4962–4970 32419290 10.1002/cam4.3135 PMC7367631

[12] Shen KY, Zhu Y, Xie SZ, Qin LX (2024) Immunosuppressive tumor microenvironment and immunotherapy of hepatocellular carcinoma: current status and prospectives. J Hematol Oncol 17:25 38679698 10.1186/s13045-024-01549-2 PMC11057182

[13] Hamstra DA, Rehemtulla A, Ross BD (2007) Diffusion magnetic resonance imaging: a biomarker for treatment response in oncology. J Clin Oncol 25:4104–4109 17827460 10.1200/JCO.2007.11.9610

[14] Koh DM, Collins DJ (2007) Diffusion-weighted MRI in the body: applications and challenges in oncology. AJR Am J Roentgenol 188:1622–1635 17515386 10.2214/AJR.06.1403

[15] Mannelli L, Kim S, Hajdu CH, Babb JS, Clark TW, Taouli B (2009) Assessment of tumor necrosis of hepatocellular carcinoma after chemoembolization: diffusion-weighted and contrast-enhanced MRI with histopathologic correlation of the explanted liver. AJR Am J Roentgenol 193:1044–1052 19770328 10.2214/AJR.08.1461

[16] Padhani AR, Liu G, Koh DM et al (2009) Diffusion-weighted magnetic resonance imaging as a cancer biomarker: consensus and recommendations. Neoplasia 11:102–125 19186405 10.1593/neo.81328 PMC2631136

[17] Zakaria R, Jenkinson MD, Radon M et al (2023) Immune checkpoint inhibitor treatment of brain metastasis associated with a less invasive growth pattern, higher T-cell infiltration and raised tumor ADC on diffusion weighted MRI. Cancer Immunol Immunother 72:3387–3393 37477652 10.1007/s00262-023-03499-z PMC10491542

[18] Cuccarini V, Aquino D, Gioppo A et al (2019) Advanced MRI assessment during dendritic cell immunotherapy added to standard treatment against glioblastoma. J Clin Med 8:2007 31744235 10.3390/jcm8112007 PMC6912338

[19] Bao X, Bian D, Yang X et al (2023) Multiparametric MRI for evaluation of pathological response to the neoadjuvant chemo-immunotherapy in resectable non-small-cell lung cancer. Eur Radiol 33:9182–9193 37382618 10.1007/s00330-023-09813-8

[20] Yu JI, Park HC, Lim DH et al (2014) The role of diffusion-weighted magnetic resonance imaging in the treatment response evaluation of hepatocellular carcinoma patients treated with radiation therapy. Int J Radiat Oncol Biol Phys 89:814–821 24969795 10.1016/j.ijrobp.2014.03.020

[21] Lee S, Kim SH, Hwang JA, Lee JE, Ha SY (2018) Pre-operative ADC predicts early recurrence of HCC after curative resection. Eur Radiol 29:1003–1012 30027408 10.1007/s00330-018-5642-5

[22] Barat M, Fohlen A, Cassinotto C et al (2017) One-month apparent diffusion coefficient correlates with response to radiofrequency ablation of hepatocellular carcinoma. J Magn Reson Imaging 45:1648–1658 27766709 10.1002/jmri.25521

[23] Liu X, Lu Y, Zhou W et al (2024) Chinese multidisciplinary expert consensus on immune checkpoint inhibitor-based combination therapy for hepatocellular carcinoma (2023 edition). Liver Cancer 13:355–375 39114757 10.1159/000535496 PMC11305662

[24] Liver EAftSot (2018) EASL clinical practice guidelines: management of hepatocellular carcinoma. J Hepatol 69:182–236 29628281 10.1016/j.jhep.2018.03.019

[25] von Elm E, Altman DG, Egger M, Pocock SJ, Gøtzsche PC, Vandenbroucke JP (2007) The strengthening the reporting of observational studies in epidemiology (STROBE) statement: guidelines for reporting observational studies. Lancet 370:1453–1457 18064739 10.1016/S0140-6736(07)61602-X

[26] Tingley D, Yamamoto T, Hirose K, Keele L, Imai K (2014) Mediation: R package for causal mediation analysis. J Stat Softw 59:1–3826917999

[27] Ciepła J, Smolarczyk R (2024) Tumor hypoxia unveiled: insights into microenvironment, detection tools and emerging therapies. Clin Exp Med 24:235 39361163 10.1007/s10238-024-01501-1 PMC11449960

[28] Winkler J, Abisoye-Ogunniyan A, Metcalf KJ, Werb Z (2020) Concepts of extracellular matrix remodelling in tumour progression and metastasis. Nat Commun 11:5120 33037194 10.1038/s41467-020-18794-x PMC7547708

[29] Mosca M, Nigro MC, Pagani R, De Giglio A, Di Federico A (2023) Neutrophil-to-lymphocyte ratio (NLR) in NSCLC, gastrointestinal, and other solid tumors: immunotherapy and Beyond. Biomolecules 13:1803 38136673 10.3390/biom13121803 PMC10741961

[30] Liao K, Zhang X, Liu J et al (2023) The role of platelets in the regulation of tumor growth and metastasis: the mechanisms and targeted therapy. MedComm (2020) 4:e350 37719444 10.1002/mco2.350 PMC10501337

[31] Xu L, Wang S, Wang S et al (2021) Baseline apparent diffusion coefficients: validation study of new predictor of survival in patients with unresectable hepatocellular carcinoma following chemoembolization. J Xray Sci Technol 29:507–516 33814481 10.3233/XST-200827

[32] Gu KW, Kim CK, Choi CH, Yoon YC, Park W (2019) Prognostic value of ADC quantification for clinical outcome in uterine cervical cancer treated with concurrent chemoradiotherapy. Eur Radiol 29:6236–6244 30980126 10.1007/s00330-019-06204-w

[33] King AD, Chow KK, Yu KH et al (2013) Head and neck squamous cell carcinoma: diagnostic performance of diffusion-weighted MR imaging for the prediction of treatment response. Radiology 266:531–538 23151830 10.1148/radiol.12120167

[34] Padhani AR, Miles KA (2010) Multiparametric imaging of tumor response to therapy. Radiology 256:348–364 20656830 10.1148/radiol.10091760

[35] Patterson DM, Padhani AR, Collins DJ (2008) Technology insight: water diffusion MRI—a potential new biomarker of response to cancer therapy. Nat Clin Pract Oncol 5:220–233 18301415 10.1038/ncponc1073

[36] Takaki S, Kurosaki M, Mori N et al (2023) Effects on survival of the adverse event of atezolizumab plus bevacizumab for hepatocellular carcinoma: a multicenter study by the Japan Red Cross Liver Study Group. Invest New Drugs 41:340–349 36995548 10.1007/s10637-023-01349-4

[37] Zhu HF, Feng JK, Xiang YJ et al (2023) Combination of alpha-fetoprotein and neutrophil-to-lymphocyte ratio to predict treatment response and survival outcomes of patients with unresectable hepatocellular carcinoma treated with immune checkpoint inhibitors. BMC Cancer 23:547 37322411 10.1186/s12885-023-11003-0 PMC10268526

[38] Smith AK, Ropella GEP, McGill MR et al (2020) Contrasting model mechanisms of alanine aminotransferase (ALT) release from damaged and necrotic hepatocytes as an example of general biomarker mechanisms. PLoS Comput Biol 16:e1007622 32484845 10.1371/journal.pcbi.1007622 PMC7292418

[39] Hanif H, Ali MJ, Susheela AT et al (2022) Update on the applications and limitations of alpha-fetoprotein for hepatocellular carcinoma. World J Gastroenterol 28:216–229 35110946 10.3748/wjg.v28.i2.216 PMC8776528

