# Supplementary material for: Mediation and prognostic value of apparent diffusion coefficient in HCC patients receiving immune checkpoint inhibitors
Source: Insights Imaging. 2025 Oct 25;16:226. doi: 10.1186/s13244-025-02115-1 (PMC12553662; doi:10.1186/s13244-025-02115-1)
Supplement: Supplementary file 1 — ELECTRONIC SUPPLEMENTARY MATERIAL [file 13244_2025_2115_MOESM1_ESM.pdf]

Mediation and prognostic value of apparent diffusion coefficient in HCC patients receiving immune checkpoint inhibitors

ELECTRONIC SUPPLEMENTARY MATERIAL

Table S1 Scan protocols for magnetic resonance imaging

		**3.0T Magnetom Skyra**	**3.0T Magnetom Verio**
T1WI	TR (ms)	3.55	3.92
	TE (ms)	1.3	1.39
	flip angle (°)	9	9
	FOV (mm)	380×309	380×285
	matrix	320×240	320×224
	thickness (mm)	3	3
	acquisition time (s)	14	19
T2WI	TR (ms)	600	800
	TE (ms)	95	61
	flip angle (°)	100	134
	FOV (mm^2)	380×297	360×360
	matrix	320×259	320×256
	thickness (mm)	6	7
	acquisition time (s)	17	18
DWI	TR (ms)	5400	4100
	TE (ms)	49	83
	PAT factor	3	2
	FOV (mm^2)	380×327	320×240
	matrix	134×134	192×154
	thickness (mm)	6	6
	acquisition time (s)	69	62

Abbreviations: TR, repetition time; TE, echo time; FOV, field of view; PAT, parallel acquisition technique

Table S21 Mediation analysis for the associations between tumor maximum diameter and NLR

Independent variable	Mediator	Total effect		Indirect effect		Direct effect		Proportion mediated, % (95% CI)
		Coefficient (95% CI)	*P* value	Coefficient (95% CI)	*P* value	Coefficient (95% CI)	*P* value	
tumor maximum diameter	wADC	0.103 (0.030, 0.180)	**0.004**	0.006 (-0.002, 0.020)	0.162	0.097 (0.026, 0.170)	**0.007**	5.6 (-2.4, 23.0)
tumor maximum diameter	sADC	0.103 (0.032, 0.180)	**0.004**	0.014 (0.002, 0.030)	**0.016**	0.089 (0.016, 0.160)	**<0.001**	13.6 (1.9, 48)

The mediation analyses were adjusted for age and sex. Abbreviations: NLR, Neutrophil to lymphocyte ratio; wADC, whole tumor apparent diffusion coefficient; sADC, substantial tumor apparent diffusion coefficient; CI, confidence interval

Table S3 Mediation analysis for the associations between tumor maximum diameter and PLR

Independent variable	Mediator	Total effect		Indirect effect		Direct effect		Proportion mediated, % (95% CI)
		Coefficient (95% CI)	*P* value	Coefficient (95% CI)	*P* value	Coefficient (95% CI)	*P* value	
tumor maximum diameter	wADC	5.242(3.082, 7.420)	**<0.001**	0.231 (-0.076, 0.65)	0.160	5.011 (2.957, 7.180)	**<0.001**	4.4 (-1.6, 13)
tumor maximum diameter	sADC	5.242(3.082, 7.420)	**<0.001**	0.468 (0.037, 1.030)	**0.033**	4.774 (2.724, 6.890)	**<0.001**	8.9 (0.8, 20)

The mediation analyses were adjusted for age and sex. Abbreviations: PLR, Platelet to lymphocyte ratio; wADC, whole tumor apparent diffusion coefficient; sADC, substantial tumor apparent diffusion coefficient; CI, confidence interval

Table S4 Tumor response between the Lower wADC and Higher wADC groups

Tumor response	Lower wADC (n=128)	Higher wADC (n=127)	*P* value
CR, n (%)	0 (0.0%)	0 (0.0%)	
PR, n (%)	32 (25%)	33 (26%)	
SD, n (%)	65 (50.8%)	71 (55.9%)	
PD, n (%)	31 (24.2%)	23 (18.1%)	
ORR, n (%)	32 (25%)	33 (26%)	0.857
DCR, n (%)	97 (75.8%)	104 (81.9%)	0.233

Abbreviations: wADC, whole tumor apparent diffusion coefficient; CR, complete response; PR, partial response; SD, stable disease; PD, progressive disease; ORR, objective response rate; DCR, disease control rate

Table S5 Tumor response between the Lower sADC and Higher sADC groups

Tumor response	Lower sADC (n=128)	Higher sADC (n=127)	*P* value
CR, n (%)	0 (0.0%)	0 (0.0%)	
PR, n (%)	30 (23.4%)	35 (27.6%)	
SD, n (%)	63 (49.2%)	73 (57.5%)	
PD, n (%)	35 (27.3%)	19 (15%)	
ORR, n (%)	30 (23.4%)	35 (27.6%)	0.450
DCR, n (%)	93 (72.7%)	108 (85%)	**0.016**

Abbreviations: sADC, substantial tumor apparent diffusion coefficient; CR, complete response; PR, partial response; SD, stable disease; PD, progressive disease; ORR, objective response rate; DCR, disease control rate

Table S6 Tumor response between the ΔwADC non-Positive and ΔwADC Positive groups

Tumor response	ΔwADC non-Positive (n=81)	ΔwADC Positive (n=87)	*P* value
CR, n (%)	0 (0.0%)	0 (0.0%)	
PR, n (%)	24 (29.6%)	16 (18.4%)	
SD, n (%)	37 (45.7%)	55 (63.2%)	
PD, n (%)	20 (24.7%)	16 (18.4%)	
ORR, n (%)	24 (29.6%)	16 (18.4%)	0.087
DCR, n (%)	61 (75.3%)	71 (81.6%)	0.320

Abbreviations: ΔwADC, relative change in whole tumor apparent diffusion coefficient; CR, complete response; PR, partial response; SD, stable disease; PD, progressive disease; ORR, objective response rate; DCR, disease control rate

Table S7 Tumor response between the ΔsADC non-Positive and ΔsADC Positive groups

Tumor response	ΔsADC non-Positive (n=82)	ΔsADC Positive (n=86)	*P* value
CR, n (%)	0 (0.0%)	0 (0.0%)	
PR, n (%)	11 (13.4%)	29 (33.7%)	
SD, n (%)	45 (54.9%)	47 (54.7%)	
PD, n (%)	26 (31.7%)	10 (11.6%)	
ORR, n (%)	11 (13.4%)	29 (33.7%)	**0.002**
DCR, n (%)	56 (68.3%)	76 (88.4%)	**0.002**

Abbreviations: ΔsADC, relative change in substantial tumor apparent diffusion coefficient**;** CR, complete response; PR, partial response; SD, stable disease; PD, progressive disease; ORR, objective response rate; DCR, disease control rate

Table S8 Univariate and multivariate Cox proportional hazards analyses for PFS in baseline total patients

Characteristics	Univariate analysis		Multivariate analysis	
	Hazard ratio (95% CI)	*P* value	Hazard ratio (95% CI)	*P* value
Age (years)	0.993 (0.981 - 1.006)	0.303		
Sex				
Female	Reference			
Male	0.731 (0.485 - 1.102)	0.134		
BMI（kg/m^2)	1.020 (0.971 - 1.071)	0.430		
Types of ICIs				
PD-1	Reference		Reference	
PD-L1	0.555 (0.283 - 1.089)	0.087	0.520 (0.264 - 1.026)	0.059
Tumor maximum diameter (cm)	1.038 (1.005 - 1.072)	**0.026**	1.031 (0.996 - 1.068)	0.085
Tumor number				
≤3	Reference			
>3	1.027 (0.739 - 1.428)	0.872		
PVTT				
No	Reference			
Yes	1.236 (0.921 - 1.661)	0.159		
Lymph node metastasis				
No	Reference			
Yes	1.208 (0.876 - 1.667)	0.249		
BCLC stage				
B	Reference		Reference	
C	1.623 (1.161 - 2.267)	**0.005**	1.229 (0.853 - 1.771)	0.269
Child-Pugh				
A	Reference			
B	1.160 (0.753 - 1.787)	0.500		
ECOG PS				
0	Reference			
≥1	1.236 (0.888 - 1.721)	0.210		
HBV infection				
No	Reference			
Yes	0.878 (0.655 - 1.178)	0.386		
Smoking				
No	Reference		Reference	
Yes	1.466 (1.057 - 2.033)	**0.022**	1.412 (1.016 - 1.963)	**0.040**
Drinking				
No	Reference			
Yes	1.044 (0.692 - 1.574)	0.838		
NLR	0.994 (0.933 - 1.059)	0.853		
PLR	1.000 (0.998 - 1.002)	0.947		
ALBI	1.193 (0.861 - 1.652)	0.289		
Hemoglobin (g/L)	0.993 (0.985 - 1.002)	0.155		
ALT(U/L)	1.001 (0.999 - 1.004)	0.414		
AST(U/L)	1.000 (0.999 - 1.001)	0.353		
AFP level (ng/ml)				
<400	Reference		Reference	
≥400	1.805 (1.340 - 2.431)	**< 0.001**	1.678 (1.227 - 2.294)	**0.001**
wADC group				
Lower wADC	Reference			
Higher wADC	0.855 (0.637 - 1.146)	0.294		
sADC group				
Lower sADC	Reference		Reference	
Higher sADC	0.699 (0.520 - 0.940)	**0.018**	0.827 (0.606 - 1.128)	0.231

Abbreviations: PFS, progression-free survival; BMI, body mass index; ICIs, immune checkpoint inhibitors; PVTT, portal vein tumor thrombosis; BCLC, Barcelona Clinic Liver Cancer; ECOG PS, Eastern Cooperative Oncology Group Performance Status; HBV infection, hepatitis B virus infection; NLR, Neutrophil to lymphocyte ratio; PLR, Platelet to lymphocyte ratio; ALBI, Albumin-Bilirubin, ALT, Alanine transaminase, AST, Aspartate transaminase; AFP, alpha-fetoprotein, wADC, whole tumor apparent diffusion coefficient; sADC, substantial tumor apparent diffusion coefficient; CI, confidence interval

Table S9 Univariate and multivariate Cox proportional hazards analyses for OS in baseline total patients

Characteristics	Univariate analysis		Multivariate analysis	
	Hazard ratio (95% CI)	*P* value	Hazard ratio (95% CI)	*P* value
Age (years)	0.996 (0.980 - 1.012)	0.621		
Sex				
Female	Reference			
Male	1.440 (0.850 - 2.441)	0.175		
BMI（kg/m^2)	1.001 (0.945 - 1.061)	0.962		
Types of ICIs				
PD-1	Reference			
PD-L1	0.485 (0.198 - 1.188)	0.113		
Tumor maximum diameter (cm)	1.055 (1.014 - 1.097)	**0.008**	1.051 (1.006 - 1.097)	**0.024**
Tumor number				
≤3	Reference			
>3	0.849 (0.570 - 1.267)	0.423		
PVTT				
No	Reference		Reference	
Yes	1.386 (0.974 - 1.972)	**0.070**	1.017 (0.705 - 1.466)	0.928
Lymph node metastasis				
No	Reference			
Yes	0.769 (0.512 - 1.154)	0.204		
BCLC stage				
B	Reference			
C	1.406 (0.927 - 2.133)	0.109		
Child-Pugh				
A	Reference			
B	1.236 (0.729 - 2.095)	0.432		
ECOG PS				
0	Reference		Reference	
≥1	1.871 (1.247 - 2.808)	**0.002**	1.967 (1.296 - 2.986)	**0.001**
HBV infection				
No	Reference			
Yes	1.019 (0.716 - 1.450)	0.918		
Smoking				
No	Reference			
Yes	1.187 (0.797 - 1.769)	0.398		
Drinking				
No	Reference			
Yes	0.705 (0.421 - 1.178)	0.182		
NLR	0.993 (0.923 - 1.068)	0.850		
PLR	1.000 (0.998 - 1.003)	0.785		
ALBI	1.240 (0.827 - 1.860)	0.298		
Hemoglobin (g/L)	0.996 (0.986 - 1.005)	0.384		
ALT(U/L)	1.005 (1.002 - 1.007)	**< 0.001**	1.006 (1.002 - 1.011)	**0.004**
AST(U/L)	1.001 (1.000 - 1.002)	**0.029**	0.999 (0.997 - 1.001)	0.198
AFP level (ng/ml)				
<400	Reference		Reference	
≥400	2.176 (1.528 - 3.100)	**< 0.001**	2.144 (1.473 - 3.121)	**< 0.001**
wADC group				
Lower wADC	Reference			
Higher wADC	0.771 (0.542 - 1.096)	0.147		
sADC group				
Lower sADC	Reference		Reference	
Higher sADC	0.509 (0.356 - 0.729)	**< 0.001**	0.626 (0.429 - 0.911)	**0.015**

Abbreviations: OS, overall survival; BMI, body mass index; ICIs, immune checkpoint inhibitors; PVTT, portal vein tumor thrombosis; BCLC, Barcelona Clinic Liver Cancer; ECOG PS, Eastern Cooperative Oncology Group Performance Status; HBV infection, hepatitis B virus infection; NLR, Neutrophil to lymphocyte ratio; PLR, Platelet to lymphocyte ratio; ALBI, Albumin-Bilirubin, ALT, Alanine transaminase, AST, Aspartate transaminase; AFP, alpha-fetoprotein, wADC, whole tumor apparent diffusion coefficient; sADC, substantial tumor apparent diffusion coefficient; CI, confidence interval

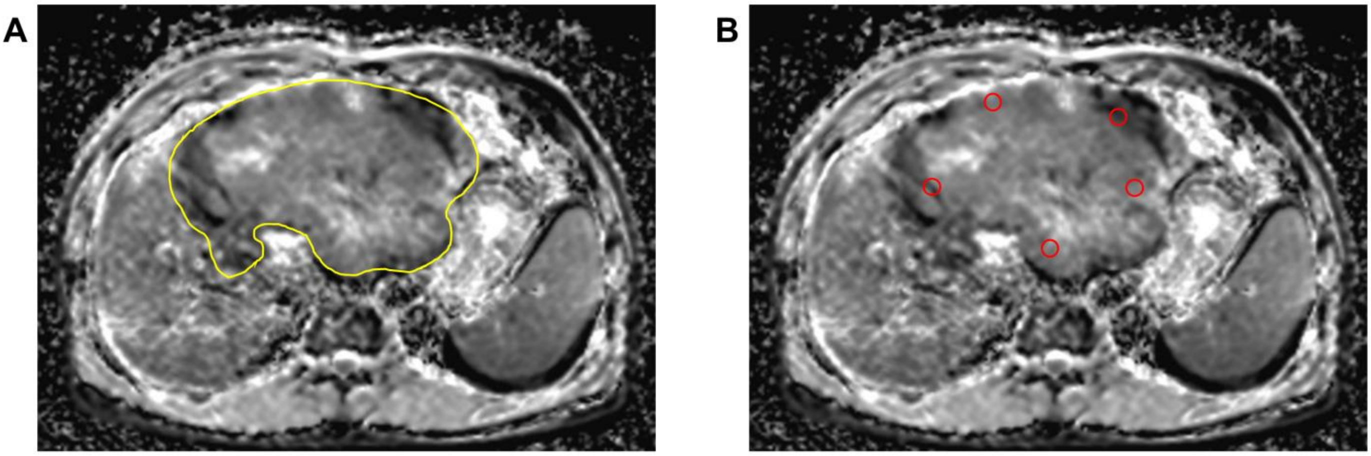

Fig. S1 Location diagrams of wADC (A) and sADC (B). wADC, whole tumor apparent diffusion coefficient; sADC, substantial tumor apparent diffusion coefficient

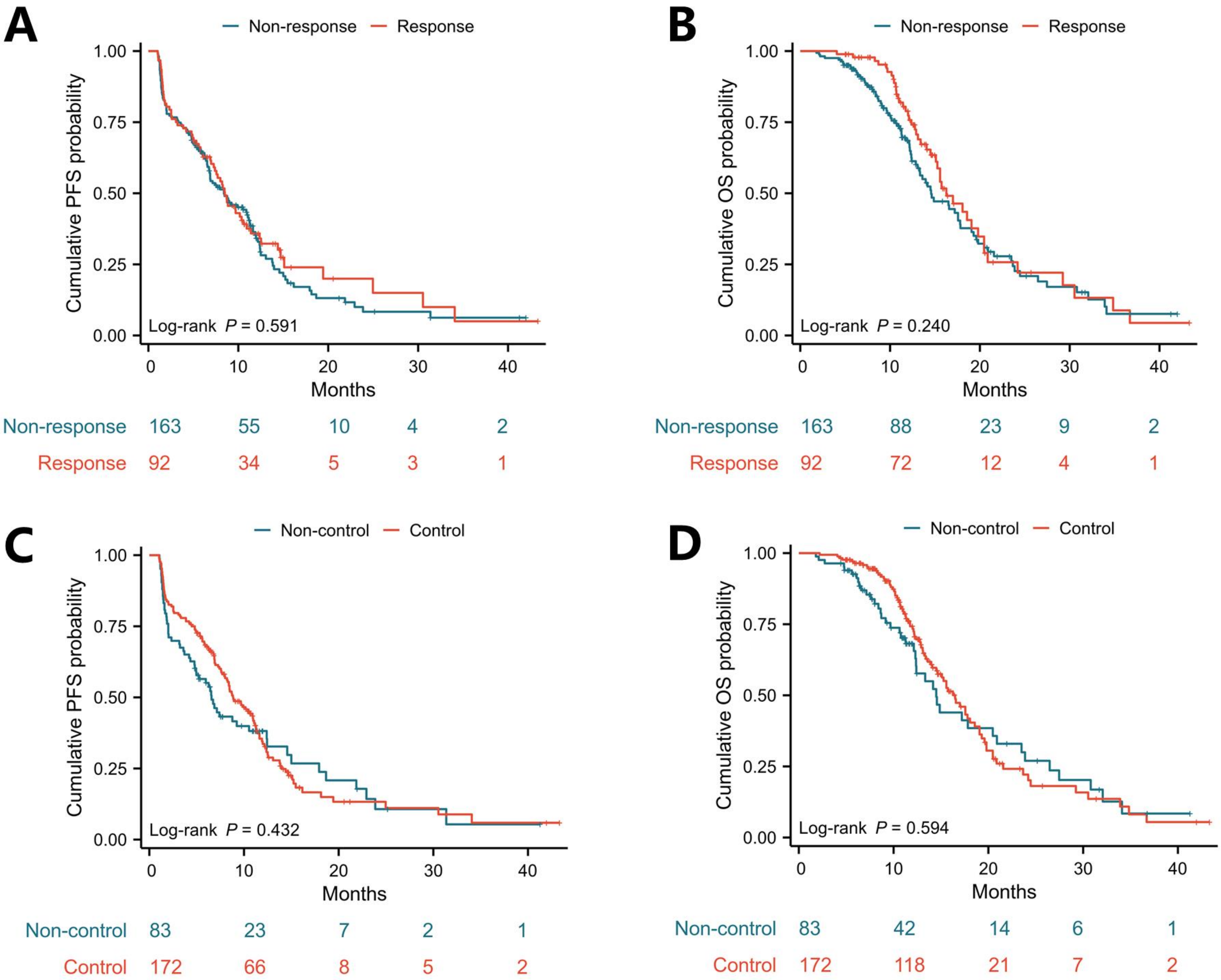

Fig. S2 Kaplan- Meier curves of wADC for PFS (A, C) and OS (B, D) in the Non-response and Response groups, as well as for the Non-control and Control groups. PFS, progression-free survival; OS, overall survival; wADC, whole tumor apparent diffusion coefficient

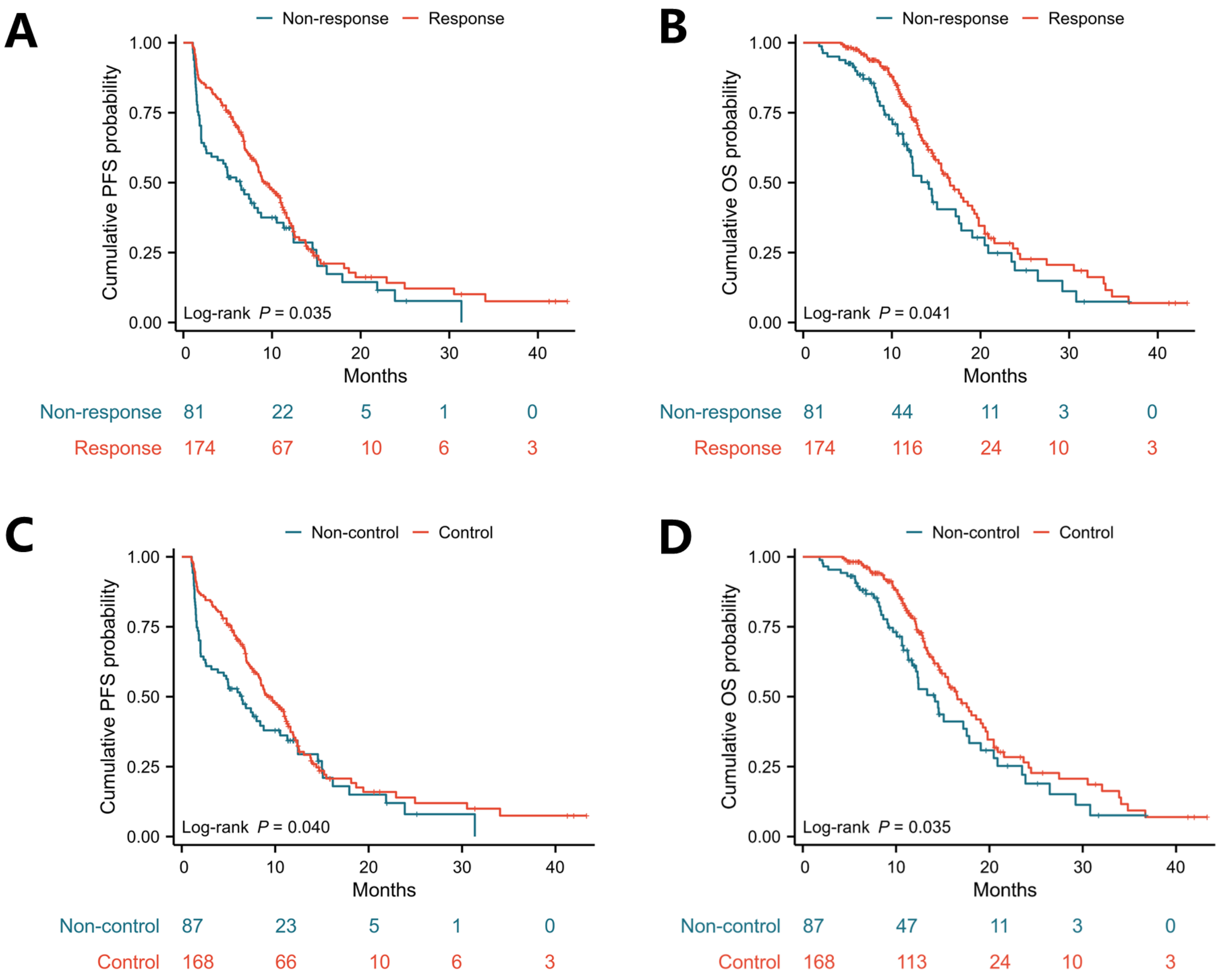

Fig. S3 Kaplan- Meier curves of sADC for PFS (A, C) and OS (B, D) in the Non-response and Response groups, as well as for the Non-control and Control groups. PFS, progression-free survival; OS, overall survival; sADC, substantial tumor apparent diffusion coefficient

Subgroup	Lower wADC	Higher wADC	HR (95% CI)
	no.		
Age (years)			
<60	76	77	0.83 (0.58, 1.19)
≥60	52	50	0.90 (0.55, 1.49)
Sex			
Female	16	18	1.22 (0.55, 2.69)
Male	112	109	0.80 (0.58, 1.10)
Types of ICIs			
PD1	121	120	0.86 (0.64, 1.16)
PD-L1	7	7	0.89 (0.22, 3.57)
Tumor number			
≤3	93	93	0.93 (0.66, 1.32)
>3	35	34	0.69 (0.38, 1.24)
PVTT			
No	76	76	0.81 (0.54, 1.21)
Yes	52	51	0.84 (0.54, 1.31)
Lymph node metastasis			
No	86	102	0.87 (0.60, 1.24)
Yes	42	25	0.80 (0.46, 1.40)
BCLC stage			
B	41	45	0.86 (0.47, 1.57)
C	87	82	0.83 (0.59, 1.17)
Child-Pugh			
A	107	112	0.82 (0.60, 1.12)
B	21	15	1.13 (0.50, 2.55)
ECOG PS			
0	88	90	0.86 (0.61, 1.22)
≥1	40	37	0.92 (0.53, 1.60)
HBV infection			
No	60	58	0.86 (0.56, 1.32)
Yes	68	69	0.84 (0.56, 1.25)
Smoking			
No	98	95	0.76 (0.54, 1.08)
Yes	30	32	1.14 (0.65, 2.01)
Drinking			
No	110	109	0.76 (0.55, 1.05)
Yes	18	18	1.32 (0.60, 2.91)
AFP level (ng/mL)			
<400	77	84	1.07 (0.72, 1.58)
≥400	51	43	0.62 (0.39, 0.98)

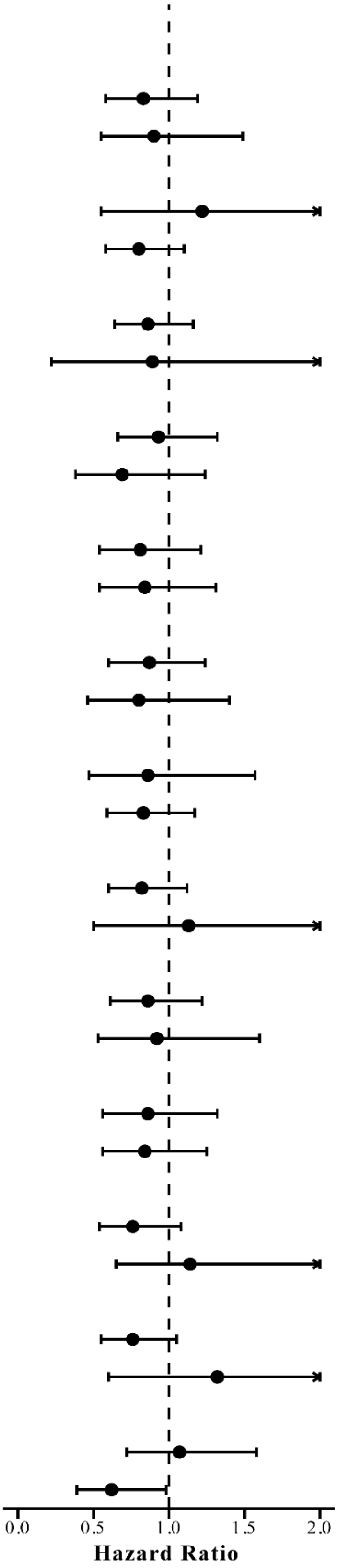

Fig. S4 Forest plot of subgroup analysis of progression-free survival between Lower wADC and Higher wADC groups. The dashed line indicates a risk ratio of 1. ICIs, immune checkpoint inhibitors; PVTT, portal vein tumor thrombosis; BCLC stage, Barcelona Clinic Liver Cancer stage; ECOG PS, Eastern Cooperative Oncology Group Physical Status; HBV, hepatitis B virus; AFP, alpha-fetoprotein; wADC, whole tumor apparent diffusion coefficient

Insights Imaging (2025) Fu X, Guo Y, Yang Y, et al.

Subgroup	Lower wADC	Higher wADC	HR (95% CI)
	no.		
Age (years)			
<60	76	77	0.73 (0.48, 1.13)
≥60	52	50	0.73 (0.38, 1.39)
Sex			
Female	16	18	1.40 (0.47, 4.21)
Male	112	109	0.73 (0.50, 1.06)
Types of ICIs			
PD1	121	120	0.74 (0.52, 1.06)
PD-L1	7	7	0.63 (0.10, 3.84)
Tumor number			
≤3	93	93	0.84 (0.56, 1.27)
>3	35	34	0.58 (0.29, 1.18)
PVTT			
No	76	76	0.53 (0.31, 0.89)
Yes	52	51	1.08 (0.66, 1.75)
Lymph node metastasis			
No	86	102	0.68 (0.45, 1.04)
Yes	42	25	0.80 (0.39, 1.64)
BCLC stage			
B	41	45	0.46 (0.21, 1.00)
C	87	82	0.88 (0.59, 1.32)
Child-Pugh			
A	107	112	0.75 (0.51, 1.09)
B	21	15	0.93 (0.34, 2.51)
ECOG PS			
0	88	90	0.76 (0.50, 1.15)
≥1	40	37	0.63 (0.31, 1.27)
HBV infection			
No	60	58	0.70 (0.42, 1.19)
Yes	68	69	0.83 (0.51, 1.35)
Smoking			
No	98	95	0.61 (0.40, 0.93)
Yes	30	32	1.13 (0.54, 2.39)
Drinking			
No	110	109	0.64 (0.44, 0.95)
Yes	18	18	1.09 (0.41, 2.92)
AFP level (ng/mL)			
<400	77	84	0.81 (0.49, 1.34)
≥400	51	43	0.77 (0.46, 1.28)

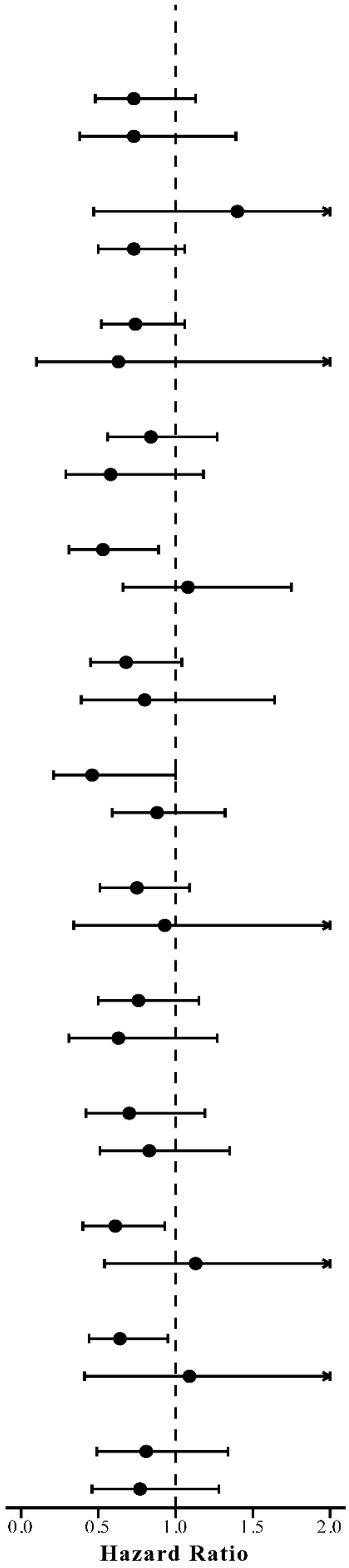

Fig. S5 Forest plot of subgroup analysis of overall survival between Lower wADC and Higher wADC groups. The dashed line indicates a risk ratio of 1. ICIs, immune checkpoint inhibitors; PVTT, portal vein tumor thrombosis; BCLC stage, Barcelona Clinic Liver Cancer stage; ECOG PS, Eastern Cooperative Oncology Group Physical Status; HBV, hepatitis B virus; AFP, alpha-fetoprotein; wADC, whole tumor apparent diffusion coefficient

Insights Imaging (2025) Fu X, Guo Y, Yang Y, et al.

Subgroup	Lower sADC	Higher sADC	HR (95% CI)
	no.		
Age (years)			
<60	80	73	0.71 (0.49, 1.02)
≥60	48	54	0.74 (0.44, 1.22)
Sex			
Female	18	16	0.59 (0.26, 1.34)
Male	110	111	0.72 (0.52, 0.99)
Types of ICIs			
PD1	122	119	0.67 (0.49, 0.90)
PD-L1	6	8	1.75 (0.42, 7.37)
Tumor number			
≤3	92	94	0.74 (0.52, 1.04)
>3	36	33	0.58 (0.31, 1.06)
PVTT			
No	66	86	0.60 (0.40, 0.90)
Yes	62	41	0.86 (0.55, 1.35)
Lymph node metastasis			
No	88	100	0.68 (0.47, 0.97)
Yes	40	27	0.77 (0.44, 1.33)
BCLC stage			
B	31	55	0.69 (0.36, 1.31)
C	97	72	0.77 (0.54, 1.09)
Child-Pugh			
A	107	112	0.66 (0.48, 0.91)
B	21	15	0.82 (0.36, 1.90)
ECOG PS			
0	89	89	0.78 (0.55, 1.11)
≥1	39	38	0.55 (0.31, 0.96)
HBV infection			
No	62	56	0.64 (0.41, 0.99)
Yes	66	71	0.77 (0.51, 1.16)
Smoking			
No	97	96	0.60 (0.42, 0.85)
Yes	31	31	1.05 (0.60, 1.84)
Drinking			
No	108	111	0.74 (0.54, 1.02)
Yes	20	16	0.47 (0.21, 1.04)
AFP level (ng/mL)			
<400	69	92	0.78 (0.53, 1.16)
≥400	59	35	0.76 (0.48, 1.22)

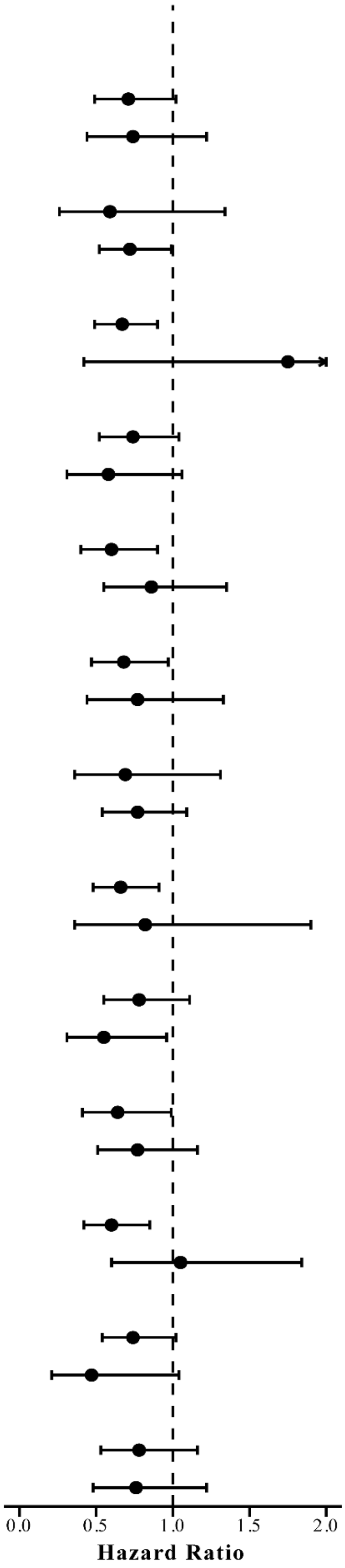

Fig. S6 Forest plot of subgroup analysis of progression-free survival between Lower sADC and Higher sADC groups. The dashed line indicates a risk ratio of 1. ICIs, immune checkpoint inhibitors; PVTT, portal vein tumor thrombosis; BCLC stage, Barcelona Clinic Liver Cancer stage; ECOG PS, Eastern Cooperative Oncology Group Physical Status; HBV, hepatitis B virus; AFP, alpha-fetoprotein; sADC, substantial tumor apparent diffusion coefficient

Subgroup	Lower sADC	Higher sADC	HR (95% CI)
	no.		
Age (years)			
<60	80	73	0.62 (0.40, 0.97)
≥60	48	54	0.31 (0.16, 0.61)
Sex			
Female	18	16	0.55 (0.18, 1.67)
Male	110	111	0.48 (0.33, 0.70)
Types of ICIs			
PD1	122	119	0.47 (0.32, 0.68)
PD-L1	6	8	0.72 (0.12, 4.36)
Tumor number			
≤3	92	94	0.55 (0.36, 0.83)
>3	36	33	0.40 (0.19, 0.83)
PVTT			
No	66	86	0.37 (0.22, 0.63)
Yes	62	41	0.72 (0.44, 1.18)
Lymph node metastasis			
No	88	100	0.45 (0.29, 0.68)
Yes	40	27	0.56 (0.27, 1.17)
BCLC stage			
B	31	55	0.36 (0.16, 0.80)
C	97	72	0.58 (0.38, 0.88)
Child-Pugh			
A	107	112	0.48 (0.33, 0.70)
B	21	15	0.66 (0.23, 1.91)
ECOG PS			
0	89	89	0.55 (0.36, 0.83)
≥1	39	38	0.40 (0.20, 0.81)
HBV infection			
No	62	56	0.42 (0.24, 0.74)
Yes	66	71	0.56 (0.35, 0.91)
Smoking			
No	97	96	0.43 (0.28, 0.66)
Yes	31	31	0.59 (0.29, 1.17)
Drinking			
No	108	111	0.49 (0.33, 0.73)
Yes	20	16	0.31 (0.10, 0.97)
AFP level (ng/mL)			
<400	69	92	0.47 (0.28, 0.77)
≥400	59	35	0.74 (0.43, 1.25)

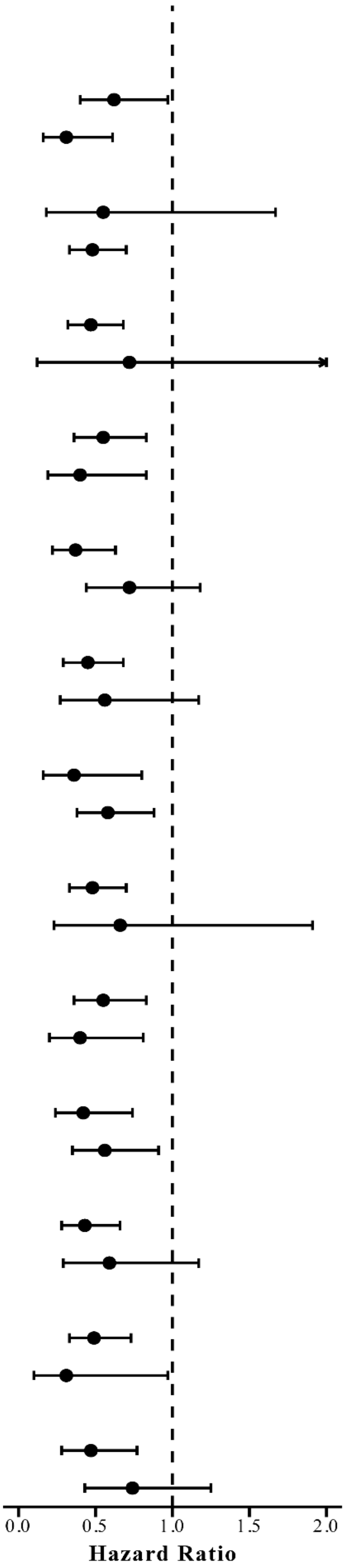

Fig. S7 Forest plot of subgroup analysis of overall survival between Lower sADC and Higher sADC groups. The dashed line indicates a risk ratio of 1. ICIs, immune checkpoint inhibitors; PVTT, portal vein tumor thrombosis; BCLC stage, Barcelona Clinic Liver Cancer stage; ECOG PS, Eastern Cooperative Oncology Group Physical Status; HBV, hepatitis B virus; AFP, alpha-fetoprotein; sADC, substantial tumor apparent diffusion coefficient

Subgroup	△wADC non-Positive	△wADC Positive	HR (95% CI)
	no.		
Age (years)			
<60	45	53	0.61 (0.38, 0.96)
≥60	36	34	0.82 (0.43, 1.56)
Sex			
Female	12	9	0.89 (0.31, 2.51)
Male	27	41	0.70 (0.47, 1.05)
Types of ICIs			
PD1	75	82	0.72 (0.49, 1.05)
PD-L1	6	5	0.41 (0.06, 2.62)
Tumor number			
≤3	63	67	0.69 (0.45, 1.05)
>3	18	20	0.84 (0.38, 1.90)
PVTT			
No	47	44	0.74 (0.44, 1.24)
Yes	34	43	0.66 (0.38, 1.14)
Lymph node metastasis			
No	59	61	0.96 (0.61, 1.50)
Yes	22	26	0.31 (0.15, 0.65)
BCLC stage			
B	26	25	0.98 (0.46, 2.09)
C	55	62	0.59 (0.38, 0.91)
Child-Pugh			
A	75	74	0.67 (0.45, 0.99)
B	6	13	1.16 (0.31, 4.41)
ECOG PS			
0	59	70	0.68 (0.44, 1.05)
≥1	22	17	0.85 (0.40, 1.79)
HBV infection			
No	38	48	1.28 (0.75, 2.19)
Yes	43	39	0.37 (0.21, 0.64)
Smoking			
No	60	68	0.76 (0.48, 1.18)
Yes	21	19	0.63 (0.31, 1.26)
Drinking			
No	67	75	0.79 (0.52, 1.20)
Yes	14	12	0.46 (0.19, 1.14)
AFP level (ng/ml)			
<400	54	54	0.68 (0.42, 1.12)
≥400	27	33	0.67 (0.37, 1.21)

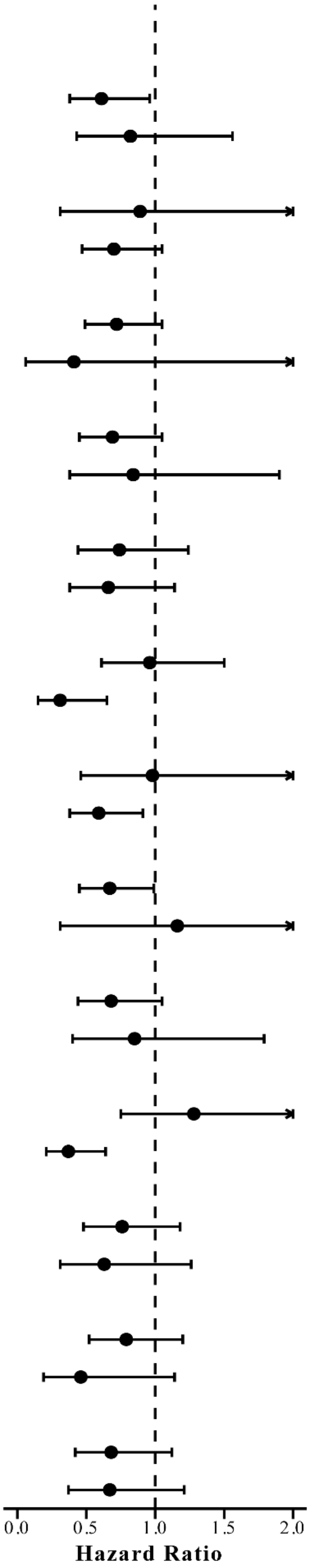

Fig. S8 ΔwADC Positive groups. The dashed line indicates a risk ratio of 1. The dashed line indicates a risk ratio of 1. ICIs, immune checkpoint inhibitors; PVTT, portal vein tumor thrombosis; BCLC stage, Barcelona Clinic Liver Cancer stage; ECOG PS, Eastern Cooperative Oncology Group Physical Status; HBV, hepatitis B virus; AFP, alpha-fetoprotein; ΔwADC, relative change in whole tumor apparent diffusion coefficient

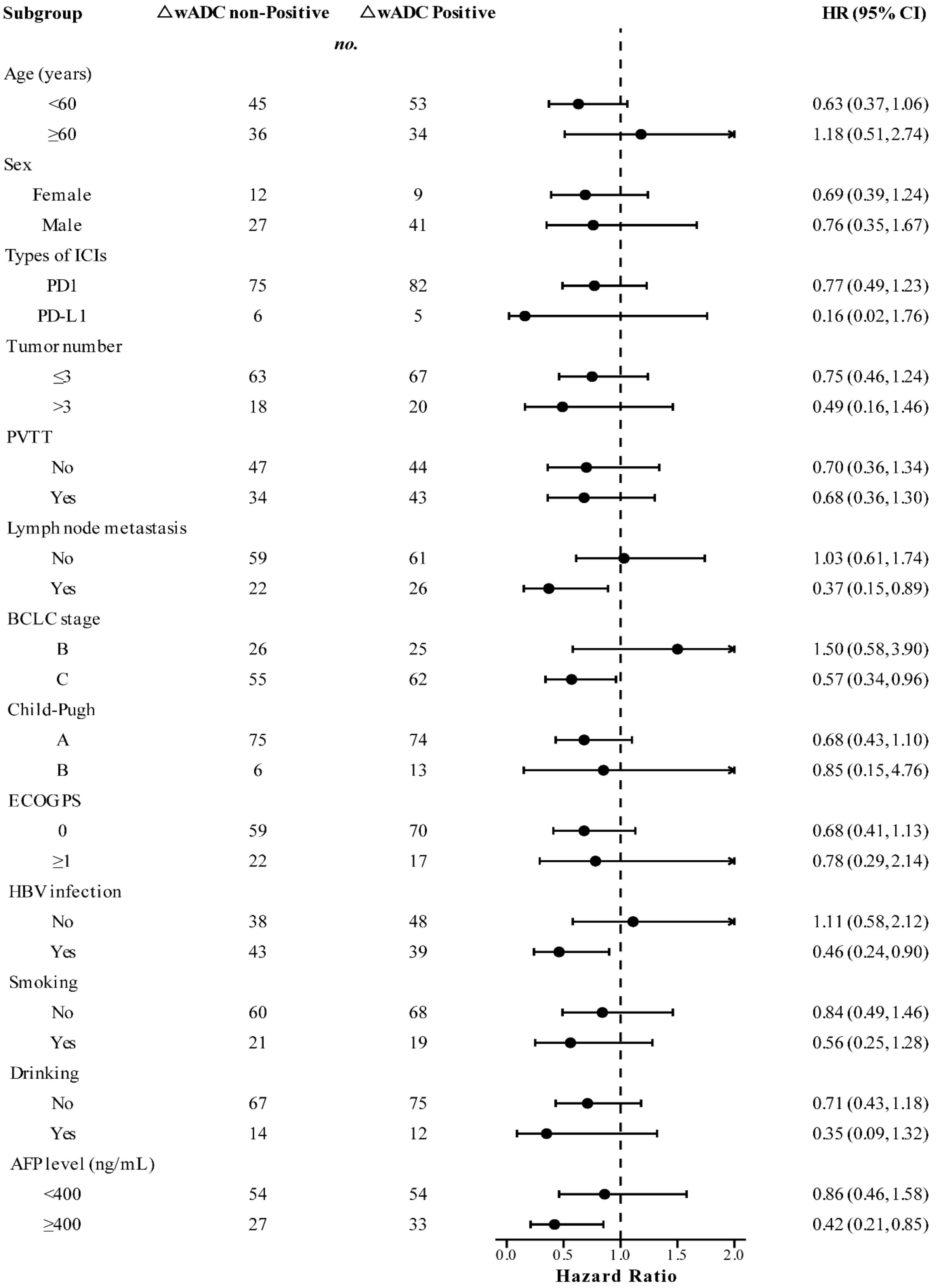

Fig. S9 Forest plot of subgroup analysis of overall survival between ΔwADC non-Positive and ΔwADC Positive groups. The dashed line indicates a risk ratio of 1. The dashed line indicates a risk ratio of 1. ICIs, immune checkpoint inhibitors; PVTT, portal vein tumor thrombosis; BCLC stage, Barcelona Clinic Liver Cancer stage; ECOG PS, Eastern Cooperative Oncology Group Physical Status; HBV, hepatitis B virus; AFP, alpha-fetoprotein; ΔwADC, relative change in whole tumor apparent diffusion coefficient

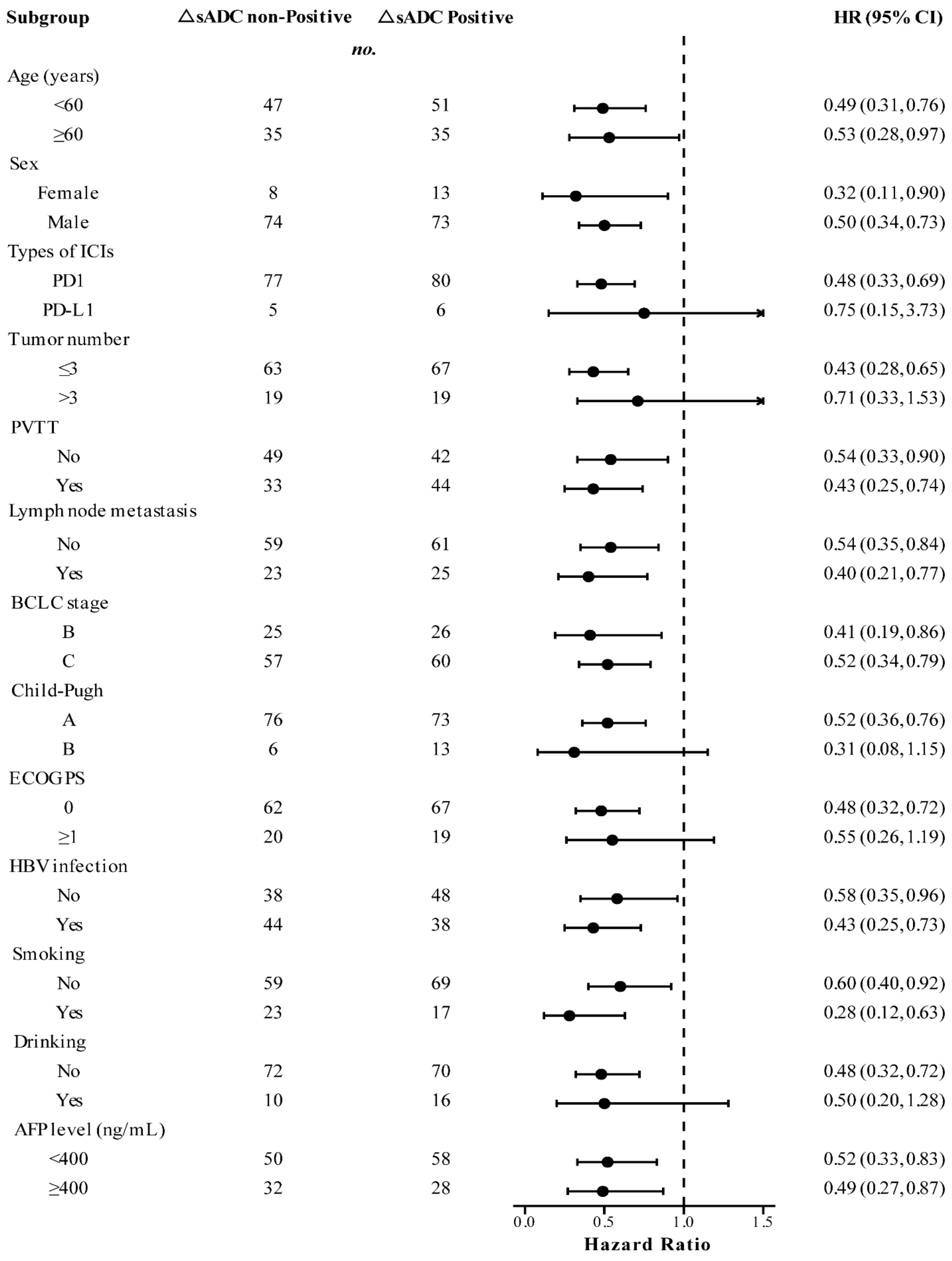

Fig. S10 Forest plot of subgroup analysis of progression-free survival between ΔsADC non-Positive and ΔsADC Positive groups. The dashed line indicates a risk ratio of 1. The dashed line indicates a risk ratio of 1. ICIs, immune checkpoint inhibitors; PVTT, portal vein tumor thrombosis; BCLC stage, Barcelona Clinic Liver Cancer stage; ECOG PS, Eastern Cooperative Oncology Group Physical Status; HBV, hepatitis B virus; AFP, alpha-fetoprotein; ΔsADC, relative change in substantial tumor apparent diffusion coefficient

Subgroup	△sADC non-Positive	△sADC Positive	HR (95% CI)
	no.		
Age (years)			
<60	47	51	0.39 (0.23, 0.66)
≥60	35	35	0.49 (0.22, 1.10)
Sex			
Female	8	13	0.28 (0.05, 1.68)
Male	74	73	0.45 (0.28, 0.72)
Types of ICIs			
PD1	77	80	0.40 (0.25, 0.64)
PD-L1	5	6	0.89 (0.15, 5.45)
Tumor number			
≤3	63	67	0.42 (0.25, 0.69)
>3	19	19	0.39 (0.14, 1.11)
PVTT			
No	49	42	0.68 (0.35, 1.31)
Yes	33	44	0.22 (0.11, 0.44)
Lymph node metastasis			
No	59	61	0.54 (0.32, 0.91)
Yes	23	25	0.25 (0.10, 0.59)
BCLC stage			
B	25	26	0.69 (0.27, 1.75)
C	57	60	0.37 (0.22, 0.62)
Child-Pugh			
A	76	73	0.46 (0.29, 0.74)
B	6	13	0.13 (0.02, 0.69)
ECOG PS			
0	62	67	0.41 (0.25, 0.68)
≥1	20	19	0.43 (0.16, 1.21)
HBV infection			
No	38	48	0.67 (0.37, 1.23)
Yes	44	38	0.24 (0.12, 0.48)
Smoking			
No	59	69	0.59 (0.35, 0.98)
Yes	23	17	0.19 (0.06, 0.59)
Drinking			
No	72	70	0.49 (0.31, 0.80)
Yes	10	16	0.26 (0.07, 0.98)
AFP level (ng/mL)			
<400	50	58	0.63 (0.35, 1.15)
≥400	32	28	0.20 (0.09, 0.44)

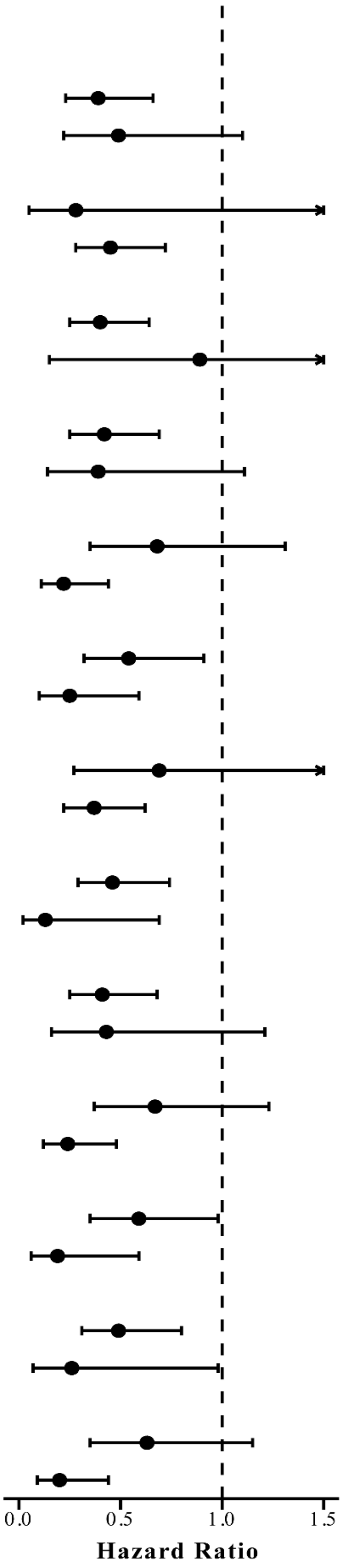

Fig. S11 Forest plot of subgroup analysis of overall survival between ΔsADC non-Positive and ΔsADC Positive groups. The dashed line indicates a risk ratio of 1. The dashed line indicates a risk ratio of 1. ICIs, immune checkpoint inhibitors; PVTT, portal vein tumor thrombosis; BCLC stage, Barcelona Clinic Liver Cancer stage; ECOG PS, Eastern Cooperative Oncology Group Physical Status; HBV, hepatitis B virus; AFP, alpha-fetoprotein; ΔsADC, relative change in substantial tumor apparent diffusion coefficient
